# Supplementary material for: Identification of a spatially organized TREM2-associated permissive macrophage niche in human pulmonary tuberculosis alveolar pneumonia
Source: Nat Commun. 2026 Aug 21;17:10817. doi: 10.1038/s41467-026-76814-8 (PMC13669344; doi:10.1038/s41467-026-76814-8)
Supplement: Supplementary file 1 [file 41467_2026_76814_MOESM10_ESM.pdf]

nature portfolio

Corresponding author(s): DARP- NCOMMS-26-016229B

Last updated by author(s): 7-5-26

# Reporting Summary

Nature Portfolio wishes to improve the reproducibility of the work that we publish. This form provides structure for consistency and transparency in reporting. For further information on Nature Portfolio policies, see our Editorial Policies and the Editorial Policy Checklist.

## Statistics

For all statistical analyses, confirm that the following items are present in the figure legend, table legend, main text, or Methods section.

| n/a | Confirmed | |
|---|---|---|
| ☐ | ☒ | The exact sample size (*n*) for each experimental group/condition, given as a discrete number and unit of measurement |
| ☐ | ☒ | A statement on whether measurements were taken from distinct samples or whether the same sample was measured repeatedly |
| ☐ | ☒ | The statistical test(s) used AND whether they are one- or two-sided<br>*Only common tests should be described solely by name; describe more complex techniques in the Methods section.* |
| ☐ | ☒ | A description of all covariates tested |
| ☐ | ☒ | A description of any assumptions or corrections, such as tests of normality and adjustment for multiple comparisons |
| ☐ | ☒ | A full description of the statistical parameters including central tendency (e.g. means) or other basic estimates (e.g. regression coefficient) AND variation (e.g. standard deviation) or associated estimates of uncertainty (e.g. confidence intervals) |
| ☐ | ☒ | For null hypothesis testing, the test statistic (e.g. *F*, *t*, *r*) with confidence intervals, effect sizes, degrees of freedom and *P* value noted<br>*Give P values as exact values whenever suitable.* |
| ☒ | ☐ | For Bayesian analysis, information on the choice of priors and Markov chain Monte Carlo settings |
| ☐ | ☒ | For hierarchical and complex designs, identification of the appropriate level for tests and full reporting of outcomes |
| ☐ | ☒ | Estimates of effect sizes (e.g. Cohen's *d*, Pearson's *r*), indicating how they were calculated |

*Our web collection on statistics for biologists contains articles on many of the points above.*

## Software and code

Policy information about availability of computer code

| | |
|---|---|
| Data collection | Spatial transcriptomic data were collected using the NanoString GeoMx Digital Spatial Profiler (DSP) platform with the Human Whole Transcriptome Atlas probe set.High-plex spatial proteomic data were generated using the NanoString CosMx Spatial Molecular Imager (SMI).Single-cell RNA sequencing libraries were prepared using the 10x Genomics Chromium Fixed RNA Profiling platform and sequenced on an Illumina NovaSeq X Plus system.Image acquisition for immunohistochemistry and immunofluorescence was performed using standard fluorescence microscopy systems.Data acquisition software was provided by NanoString Technologies and 10x Genomics according to manufacturer protocols. |
| Data analysis | Spatial transcriptomic data were processed and analyzed using GeomxTools (v3.10.0), GeoMxWorkflows (v1.12.0), NanoStringNCTools (v1.14.0), and DESeq2 (v1.46.0) in R.Single-cell RNA sequencing data were processed using the 10x Genomics Cell Ranger pipeline (v7.2.0 and v9.0.1) and analyzed using Seurat (v5.1.0) with Harmony integration.Functional enrichment analyses were performed using Metascape (v3.5).Correlation and gene set overlap analyses were performed in R.A custom Python script using NCBI E-utilities was used for automated PubMed searches of antimicrobial genes.All software used is publicly available or commercially distributed by the respective manufacturers. |

For manuscripts utilizing custom algorithms or software that are central to the research but not yet described in published literature, software must be made available to editors and reviewers. We strongly encourage code deposition in a community repository (e.g. GitHub). See the Nature Portfolio guidelines for submitting code & software for further information.

## Data

Policy information about availability of data

All manuscripts must include a data availability statement. This statement should provide the following information, where applicable:

- Accession codes, unique identifiers, or web links for publicly available datasets
- A description of any restrictions on data availability
- For clinical datasets or third party data, please ensure that the statement adheres to our policy

The spatial transcriptomic (GeoMx DSP) and single-cell RNA sequencing (scRNA-seq) datasets generated in this study are deposited in the Gene Expression Omnibus (GEO) under accession number [to be provided upon acceptance]. Processed count matrices and associated metadata will be made publicly available upon publication.Spatial proteomic (CosMx) data supporting the findings of this study will be made available in a public repository [GEO or other appropriate repository] under accession number [to be provided].Source data underlying figures and statistical analyses are provided with the paper.All other data supporting the findings of this study are available from the corresponding author upon reasonable request.

## Research involving human participants, their data, or biological material

Policy information about studies with human participants or human data. See also policy information about sex, gender (identity/presentation), and sexual orientation and race, ethnicity and racism.

| | |
|---|---|
| Reporting on sex and gender | Human pulmonary tuberculosis biopsy specimens and peripheral blood from healthy adult donors were obtained under institutional review board–approved protocols with written informed consent. Sex of PTB participants is reported in Supplementary Table 1. Sex and gender were not considered as biological variables in in vitro macrophage experiments, as the study was not designed or powered to assess sex-based differences. |
| Reporting on race, ethnicity, or other socially relevant groupings | Race and ethnicity information were not collected for this study, as they were not relevant to the mechanistic objectives of the investigation. |
| Population characteristics | The study included four adult patients with untreated pulmonary tuberculosis undergoing diagnostic lung biopsy at Charles Nicolle Hospital (Tunis, Tunisia). All patients were enrolled prior to initiation of anti-tuberculous therapy. Additional in vitro experiments were performed using peripheral blood mononuclear cells obtained from healthy adult donors. |
| Recruitment | Patients presenting with suspected pulmonary tuberculosis requiring diagnostic lung biopsy at Charles Nicolle Hospital (Tunis, Tunisia) were recruited at the time of clinical evaluation prior to initiation of therapy. Healthy adult blood donors were recruited under institutional review board–approved protocols for research blood donation. |
| Ethics oversight | The study was conducted in accordance with the Declaration of Helsinki and was approved by the Institut Pasteur de Tunis Institutional Review Board (2021/26/I/V1) and the Charles Nicolle Hospital Institutional Review Board (2022/06/EC/V1). All participants provided written informed consent prior to sample collection. |

Note that full information on the approval of the study protocol must also be provided in the manuscript.

# Field-specific reporting

Please select the one below that is the best fit for your research. If you are not sure, read the appropriate sections before making your selection.

☒ Life sciences ☐ Behavioural & social sciences ☐ Ecological, evolutionary & environmental sciences

For a reference copy of the document with all sections, see nature.com/documents/nr-reporting-summary-flat.pdf

# Life sciences study design

All studies must disclose on these points even when the disclosure is negative.

| | |
|---|---|
| Sample size | The study included pulmonary biopsy specimens from four independent patients with untreated tuberculosis. Sample size was determined by availability of diagnostic specimens containing both alveolar pneumonia and granulomas. No formal statistical power calculation was performed. In vitro experiments were conducted using macrophages derived from multiple independent healthy donors, with the number of donors indicated in the corresponding figure legends. |
| Data exclusions | Regions of interest failing predefined NanoString quality control criteria were excluded prior to analysis. One ROI with partial tissue contamination was removed from region-specific comparisons. For single-cell RNA sequencing, low-quality nuclei were excluded based on predefined thresholds (≥400 detected genes and ≤5% mitochondrial RNA). No other data were excluded. |
| Replication | Spatial transcriptomic findings were validated using independent complementary approaches, including single-cell RNA sequencing, spatial proteomics, immunohistochemistry, and in situ hybridization. In vitro macrophage experiments were performed across multiple independent healthy donors, and key findings were reproducible across donors. |
| Randomization | No randomization was performed. Human biopsy specimens were obtained based on clinical indication, and in vitro experimental conditions |

| | |
|---|---|
| Randomization | were applied uniformly across samples |
| Blinding | No blinding was performed. Tissue regions were selected based on established histological criteria, and computational analyses were conducted using standardized automated pipelines. |

# Reporting for specific materials, systems and methods

We require information from authors about some types of materials, experimental systems and methods used in many studies. Here, indicate whether each material, system or method listed is relevant to your study. If you are not sure if a list item applies to your research, read the appropriate section before selecting a response.

## Materials & experimental systems

| n/a | Involved in the study |
|---|---|
| ☐ | ☒ Antibodies |
| ☒ | ☐ Eukaryotic cell lines |
| ☒ | ☐ Palaeontology and archaeology |
| ☒ | ☐ Animals and other organisms |
| ☐ | ☒ Clinical data |
| ☒ | ☐ Dual use research of concern |
| ☒ | ☐ Plants |

## Methods

| n/a | Involved in the study |
|---|---|
| ☒ | ☐ ChIP-seq |
| ☐ | ☒ Flow cytometry |
| ☒ | ☐ MRI-based neuroimaging |

## Antibodies

| | |
|---|---|
| Antibodies used | The following antibodies were used in this study: anti-TREM2 (APC-conjugated, rat IgG2b, R&D Systems, Cat# FAB17291A) and anti-CD163 (PerCP-eFluor 710, mouse IgG1, Thermo Fisher Scientific, Cat# 46-1639-42) for flow cytometry; anti-CD3 (rat IgG1, Bio-Rad, Cat# MCA1477), anti-CD68 (mouse IgG1, Thermo Fisher Scientific, Cat# 14-0688-82), anti-CD20 (mouse IgG2a, Thermo Fisher Scientific, Cat# 14-0202-37), anti-CD4 (rabbit IgG, Abcam, Cat# ab133616), anti-CD8 (mouse IgG1, Agilent Dako, Cat# M7103), anti-CD163 (mouse IgG2a, Lifespan Biosciences), anti-TREM2 (mouse IgG3, Novus Biologicals, Cat# NBP1-07101), anti-TREM2 Alexa Fluor 488–conjugated (Novus Biologicals, Cat# NBP1-07101AF488), anti-PLIN2/ADRP (mouse IgG1, Thermo Fisher Scientific, Cat# MA5-24797), anti-APOE (mouse IgG1, Novus Biologicals, Cat# NB110-60531), and anti-SPP1/osteopontin (mouse IgG1, Novus Biologicals, Cat# NB110-89062) for immunohistochemistry and immunofluorescence analyses. |
| Validation | All antibodies used in this study were commercially available and validated by the manufacturers for the indicated applications. Antibodies were titrated and validated in control human tonsil and lymph node tissues prior to experimental use. Specificity was confirmed by appropriate staining patterns consistent with known cellular localization and tissue distribution. For flow cytometry, matched isotype controls were included |

## Clinical data

Policy information about clinical studies
All manuscripts should comply with the ICMJE guidelines for publication of clinical research and a completed CONSORT checklist must be included with all submissions.

| | |
|---|---|
| Clinical trial registration | No clinical trial was conducted |
| Study protocol | No clinical trial protocol applies, as this study is an observational analysis of human biopsy specimens and in vitro experiments. |
| Data collection | Clinical data and biopsy specimens were collected at the time of diagnostic evaluation for suspected pulmonary tuberculosis, prior to initiation of anti-tuberculous therapy. Tissue samples were obtained as part of standard clinical care, and no experimental interventions were performed. |
| Outcomes | No predefined clinical outcomes were assessed, as this study was not designed as a clinical trial. The investigation focused on molecular and cellular characterization of biopsy specimens and mechanistic in vitro experiments rather than patient outcome measures. |

## Plants

| | |
|---|---|
| Seed stocks | This study did not involve plants or plant-derived materials. |
| Novel plant genotypes | This study did not involve plants or plant-derived materials. |
| Authentication | This study did not involve plants or plant-derived materials. |

## Flow Cytometry

### Plots

Confirm that:

☒ The axis labels state the marker and fluorochrome used (e.g. CD4-FITC).

☒ The axis scales are clearly visible. Include numbers along axes only for bottom left plot of group (a 'group' is an analysis of identical markers).

☒ All plots are contour plots with outliers or pseudocolor plots.

☒ A numerical value for number of cells or percentage (with statistics) is provided.

### Methodology

| | |
|---|---|
| Sample preparation | Monocyte-derived macrophages were harvested, washed in phosphate-buffered saline, and incubated with fluorochrome-conjugated antibodies against surface markers according to manufacturer instructions. Cells were stained in appropriate staining buffer, washed to remove unbound antibody, and resuspended prior to acquisition. Where indicated, isotype controls were included. Samples were kept on ice and protected from light until analysis. |
| Instrument | Flow cytometry data were acquired on an Attune NxT Flow Cytometer (Thermo Fisher Scientific) according to the manufacturer's instructions. Instrument settings and compensation were established using unstained and single-stained controls prior to data acquisition. |
| Software | Flow cytometry data were analyzed using FlowJo software (version X, BD Biosciences). Gating strategies were applied uniformly across samples using unstained and isotype controls to define positive populations. |
| Cell population abundance | Cell population abundance was determined as the percentage of total live singlet cells within the gated population. Gating thresholds were defined using unstained and isotype controls and applied consistently across all samples. |
| Gating strategy | Cells were initially gated based on forward and side scatter to exclude debris, followed by singlet discrimination using FSC-A versus FSC-H parameters. Live cells were gated where applicable, and marker-positive populations were defined using unstained and isotype controls. The same gating strategy was applied consistently across all samples. |

☒ Tick this box to confirm that a figure exemplifying the gating strategy is provided in the Supplementary Information.
