# Supplementary material for: Identification of a spatially organized TREM2-associated permissive macrophage niche in human pulmonary tuberculosis alveolar pneumonia
Source: Nat Commun. 2026 Aug 21;17:10817. doi: 10.1038/s41467-026-76814-8 (PMC13669344; doi:10.1038/s41467-026-76814-8)
Supplement: Supplementary file 2 [file 41467_2026_76814_MOESM11_ESM.pdf]

nature portfolio

Peer Review File

Identification of a spatially organized TREM2-associated permissive macrophage niche in human pulmonary tuberculosis alveolar pneumonia

Corresponding Author: Dr Robert Modlin

This file contains all reviewer reports in order by version, followed by all author rebuttals in order by version.

Version 0:

Reviewer comments:

Reviewer #1

(Remarks to the Author)
The manuscript presents a solid spatial/in situ data package showing enrichment of TREM2-associated, lipid-laden macrophages in TB pneumonia alongside the more organized immune architecture of adjacent granulomas, but the main findings are presented in a largely descriptive manner.

In my view, the work is largely incremental relative to the substantial existing literature on macrophage heterogeneity in TB, granuloma biology, foamy macrophages, and TREM2-associated macrophage states, including prior work on TREM2 in M. tuberculosis infection, foamy macrophages as a nutrient-rich reservoir for M. tuberculosis persistence, and mycobacterial mycolates modulating cholesterol handling in macrophages.

The conclusions are broadly consistent with the data, but several figures remain mainly descriptive rather than strongly quantitative, and additional quantitative analysis and clearer statistical support would strengthen the claims.

I do not see major technical flaws in the methodology or analysis, and the methods appear sound and sufficiently detailed for reproduction, but the interpretation feels underdeveloped and the level of novelty is, in my opinion, insufficient to support publication in the present form.

Figures 2–5 are largely descriptive and would benefit from more quantitative analysis. In their current form, the images clearly illustrate the spatial patterns the authors want readers to notice, but the conclusions would be strengthened by additional quantification and clearer statistical support. I would therefore encourage the authors to reconsider these figures with appropriate quantification and to make the key biological contrasts more explicit.

(Remarks on code availability)

Reviewer #2

(Remarks to the Author)
This paper carries out extensive and advanced microscopic examination of different tuberculosis lesions in the biopsied lungs of persons with early tuberculosis.

The authors compare and contrast the profile and behaviour of cells that are contained in the granuloma, to the cells on the same slide, but in the adjacent ‘alveolar pneumonia’ area of the lung. In order to explore their hypothesis, they demonstrate that Mtb derived bacterial derived material is prevalent in the alveolar pneumonia, and that this material (including PDIM and mycolic acids) likely drives surface macrophage TREM 2 to accumulate, resulting in lipid droplet accumulation and supporting Mtb growth, which will be reversed with 1-25 VitD3.
The authors provide advanced staining and molecular profiling and subsequent statistical analysis of the four specimens.. over 7 data figures (22 supplemental figures); and they do spatial transcriptomic profiling, single cell RNA seq, and GeoMx spatial proteomics.

The novelty here is that the authors bring to the attention of the reader the process of inadequate host defence against TB that is adjacent to the granuloma. There is abundant indirect evidence of the presence of bacilli in that neighbourhood> This area is otherwise conducive to Mtb growth and is now also open to host targeting therapy to manipulate pathways of immune evasion.

Given that the alveoli is where surfactant metabolism happens, it's not surprising that the molecular profiling (alveolar) reflects that activity, in contrast to the granuloma region, where instead .. signatures of pro inflammatory and anti microbial genes were shown. This dichotomy of function is well demonstrated in the Jaccard analysis shown in figure 1K. I was reassured to see the SPP1 macrophages in the alveolar compartment mostly, as osteopontin has such a crucial role in granuloma structure. The TREM correlation with specific leprosy phenotypes is strong. The in-situ identification of Mtb derived material is interesting. Its prevalence in cells, albeit in the complete absence of intact bacilli, has already been investigated by some of the authors who published in E biomedicine in 2024 Nargan et al antigen 85 staining of epithelial cells (ref 46). The suggestion here, is the alveolar pneumonia regions appear to be replete with macrophages may get rid of recognizable Mtb bacilli from the slide, but without efficiently getting rid of the bacillus-derived material such as TB Lam or TB PPD, is that correct?

Some in vitro work follows where monocyte derived macrophages are analyzed before and after stimulation with IL 4 or Mtb derived reagents and examined for lipid droplet accumulation and expression of TREM2. In figure 7, the reagent 1,25 dihydroxy vitamin D is used - consistent with the track record of the authors. It would be interesting to see how all trans retinoic acid (ATRA) performs, side by side, in the same assays performed in figure 7 (as a positive control).

(Remarks on code availability)

Reviewer #3

(Remarks to the Author)
This manuscript presents an interesting spatial analysis of human pulmonary tuberculosis. The authors compare alveolar pneumonia and adjacent granulomas in the same lung biopsies from four treatment-naïve patients. Although the sample size is small, they report several intriguing findings. They show that TREM2$^+$ lipid-laden foamy macrophages dominate the alveolar compartment and create a permissive niche for Mtb persistence with very few T cells. This is in clear contrast to the organized lymphoid structures and strong antimicrobial programs in the neighboring granulomas.
PDIM and autophagy are known to be important in TB infection and have been well described in previous studies. The real highlight here is the sharp spatial separation between the lipid-droplet-rich permissive alveolar niche and the adaptive-immunity-rich granulomatous compartment. This contrast is particularly interesting and adds valuable new insight into the understudied early alveolar phase of human TB pathogenesis.
While the intra-patient comparative design is elegant and the multi-modal dataset is impressive, several major and minor issues should be addressed before the manuscript can be accepted.
Major comments:

1.The study is based on only four patients, all with sputum-negative, biopsy-required early/atypical presentations. This limits generalizability. Please add a supplemental Table 1 with detailed clinical information for each patient and discuss selection bias and generalizability in the Discussion.
2.There are no non-TB controls (healthy lung or other inflammatory lung diseases). It is hard to know whether the TREM2$^+$ foamy macrophage program is specific to TB or a general response to alveolar lipid accumulation. The authors should either add such controls or clearly tone down claims of a “TB-specific permissive niche”.
3.The mechanistic link to TREM2 is correlative. In-vitro experiments rely on IL-4 polarization, and there is no loss-of-function validation (TREM2 knockdown/knockout, blocking antibody, or DAP12 inhibitor) for PDIM/mycolic acid effects or the vitamin D rescue. These experiments are needed to establish causality. I think it would also be necessary to perform TREM2 knockout experiments using immortalized cell lines or TREM2 knockout mice.
4.Bacterial presence in tissue is inferred from RNAscope mRNA and LAM/PPD staining. Given the AFB-negative biopsies, please provide quantitative puncta counts per cell/ROI with statistics and openly discuss the limitations of these readouts (e.g., mRNA half-life, detection of non-viable organisms).
5.Some key methodological details are missing or unclear (exact ROI numbers per patient/compartment, multiple-testing correction, batch-effect handling, Jaccard index thresholds). These should be clarified in the Methods or supplement to meet current standards for spatial omics studies.
6.To directly address the highlighted relationship between lipid-droplet accumulation and impaired adaptive immunity, it would be valuable to perform co-culture experiments with TREM2$^+$ foamy macrophages and autologous T cells (before and after lipid reduction by vitamin D) to test whether lowering lipid droplets can restore T cell activation or recruitment.

Minor comments:

1.Abstract: state n=4 patients and main methods early, and quantify “sparse T-cell infiltration” where possible.
2.Several long sentences in the Introduction (especially lines 60–72) would be easier to read if split.
3.Correct the recurring typo “Mb growth” to “Mtb growth” throughout.
4.All figures and legends need scale bars, statistical annotations, and quantitative data (e.g., puncta counts, lipid droplet area, CFU) shown as bar graphs rather than representative images only.
5.Methods: provide source, purity, solvent controls, endotoxin levels and exact concentrations for PDIM and free mycolic acids, plus full details on Mtb strain, MOI and infection conditions.

6.Standardize terminology (“alveolar pneumonia” vs “alveoli” vs “pneumonia lesions”) and give a quantitative definition of “foamy macrophages” (e.g., $PLIN2^{+}$ area or Oil Red O positivity).
7.scRNA-seq: report cell numbers per patient before/after filtering, clustering resolution, and top marker genes (ideally in a supplemental table).
8.Add a Data Availability statement with GEO accession numbers (or commitment to upload upon acceptance).
9.The transmission claim is interesting but speculative; consider adding “potentially” or “may facilitate”.

(Remarks on code availability)
The GitHub repository contains relevant scripts for GeoMx, scRNA-seq, CosMx, and text mining analyses, organized into clear modules. It links to GEO and Zenodo data. However, the README provides only a high-level description and lacks detailed installation instructions, dependency lists, or step-by-step running guidelines. The code appears usable for reproducing the main analyses with some effort, but improved documentation would make it a more accessible resource for the community.

Version 1:

Reviewer comments:

Reviewer #1

(Remarks to the Author)
Thank you for the revision and for addressing the questions.

I recommend moving the quantifications of Figures 2, 3, 4, and 5 into the main body of the manuscript. This would help avoid presenting images without accompanying quantitative analysis and would strengthen the presentation of the data within the manuscript.

The remaining figures are of high quality.

The text revisions have improved the clarity of the manuscript, and the overall message is now conveyed very clearly.

I also recommend more thorough annotation and development of several supplementary figures. In particular, Supplementary Figures 1, 2, 9, 10, 11, 12, 14, 15, 16, and 18 appear somewhat underdeveloped and would benefit from additional labeling, explanatory details, and figure annotations to improve clarity and facilitate interpretation by readers.

(Remarks on code availability)
I cannot judge code

Reviewer #2

(Remarks to the Author)
satisfactory changes to my comments are complete

(Remarks on code availability)

Reviewer #3

(Remarks to the Author)
The revised manuscript is substantially improved. The authors have added useful clinical information, additional quantification, comparison with other lung disease datasets, and a clearer discussion of limitations.

My remaining concern is mainly interpretive. The data support a TREM2-positive foamy macrophage state associated with lipid accumulation and enrichment of Mtb transcripts/antigens in alveolar regions. However, without TREM2 loss-of-function, blocking, or pathway-inhibition experiments, the study does not establish that TREM2 itself is causally required for lipid accumulation, impaired autophagy, or enhanced Mtb survival.

I do not request additional experiments at this stage. I recommend that the authors make a final textual revision to moderate causal language throughout the manuscript, especially in the title, abstract, results, figure legends, and discussion. The findings should be framed as identifying a spatially organized TREM2-associated permissive macrophage niche, rather than proving a TREM2-dependent causal pathway. The vitamin D data should also be described cautiously as macrophage reprogramming associated with reduced TREM2 expression, lipid droplets, and bacterial viability, not as definitive evidence of a TREM2-specific rescue mechanism.

I also suggest that the authors ensure the figure legends and statistical descriptions clearly distinguish donor-level biological replication from ROI- or image-level measurements.

(Remarks on code availability)

I have no additional code-specific concerns.

Open Access This Peer Review File is licensed under a Creative Commons Attribution 4.0 International License, which permits use, sharing, adaptation, distribution and reproduction in any medium or format, as long as you give appropriate credit to the original author(s) and the source, provide a link to the Creative Commons license, and indicate if changes were made.
In cases where reviewers are anonymous, credit should be given to 'Anonymous Referee' and the source.
The images or other third party material in this Peer Review File are included in the article's Creative Commons license, unless indicated otherwise in a credit line to the material. If material is not included in the article's Creative Commons license and your intended use is not permitted by statutory regulation or exceeds the permitted use, you will need to obtain permission directly from the copyright holder.
To view a copy of this license, visit https://creativecommons.org/licenses/by/4.0/

Response to Reviewers

NCOMMS-26-016229A: TREM2$^+$ macrophages accumulate in alveoli of human pulmonary tuberculosis providing a permissive niche for bacterial growth

REVIEWER #1

Comment 1.1. “The manuscript presents a solid spatial/in situ data package showing enrichment of TREM2-associated, lipid-laden macrophages in TB pneumonia alongside the more organized immune architecture of adjacent granulomas, but the main findings are presented in a largely descriptive manner.”

Response: We thank the reviewer for this comment. While the study includes extensive spatial in situ data, it is not limited to descriptive analysis. The manuscript integrates functional and mechanistic experiments that directly link macrophage state to bacterial persistence. These include:
• Identification of PDIM and mycolic acids as inducers of TREM2 expression and signaling.
• Direct measurement of TREM2–DAP12 activation using a reporter system.
• Functional assays demonstrating that TREM2$^+$ foamy macrophages exhibit increased intracellular Mtb viability and reduced antimicrobial activity.
• Demonstration that this phenotype is pharmacologically reversible by vitamin D, restoring autophagy and reducing bacterial burden.

In addition, we have strengthened the spatial analysis by transforming the in situ observations into quantitatively supported findings, including:
• FOV-level statistical comparisons (alveoli vs. core vs. mantle).
• Quantification of cell-type enrichment using Jaccard indices with adjusted p-values.
• Image-based quantification (TREM2$^+$ cells, PLIN2$^+$ lipid droplets, antigen detection, and RNAscope puncta).

Together, these revisions clarify that the study integrates spatial, quantitative, and functional analyses to define a lesion-specific macrophage program linked to *Mtb* persistence. These clarifications are reflected in the revised Results and Discussion sections.

Comment 1.2. “In my view, the work is largely incremental relative to the substantial existing literature on macrophage heterogeneity in TB, granuloma biology, foamy macrophages, and TREM2-associated macrophage states, including prior work on TREM2 in M. tuberculosis infection, foamy macrophages as a nutrient-rich reservoir for M. tuberculosis persistence, and mycobacterial mycolates modulating cholesterol handling in macrophages.”

Response: We thank the reviewer for this thoughtful comment and agree that substantial prior work has established macrophage heterogeneity in tuberculosis, including foamy macrophages, TREM2-associated macrophage programs, and lipid-driven metabolic remodeling. However, these studies have largely been performed without spatial resolution or without direct comparison of distinct lesion compartments within the same human lung.

The central advance of our study is therefore not the identification of these macrophage states per se, but the demonstration that they are spatially organized into functionally distinct compartments within the same lesion. By directly comparing alveolar pneumonia and adjacent granulomas within individual patients, we show that TREM2$^+$ lipid-laden macrophages are not simply one component of heterogeneous macrophage populations, but are preferentially localized to the alveolar compartment, where they are coupled to increased Mtb transcripts and protein with reduced adaptive immune engagement.

This spatial organization reveals a previously unrecognized lesion-specific immune architecture in which macrophage programs, bacterial burden, and adaptive immune responses are compartmentalized within the same lung. The integration of spatial transcriptomics, single-cell RNA sequencing, and high-resolution imaging in human tissue enables this analysis and allows us to link macrophage state to both anatomical location and functional outcome.

We have revised the manuscript to clarify that the advance lies in defining lesion-specific spatial and functional divergence, rather than reiterating macrophage heterogeneity.

Introduction. We have added, “Critically, prior studies have not defined how these macrophage states are spatially organized within human pulmonary lesions or how they relate to bacterial localization and immune function within the same lung.”

Results. Revised the description of spatial clustering to emphasize differences between alveolar and granulomatous regions within the same lesion (Figure 1I). “Unsupervised clustering of the transcriptomes clearly segregated regions of interest by their spatial location (alveoli vs. core vs. mantle), indicating distinct molecular signatures for each compartment, with gene expression programs and immune cell composition differing markedly between alveolar and granulomatous regions within the same lesion (Figure 1I).”

Discussion. We have added a new opening paragraph: “Spatial transcriptomic and single-cell analyses of human pulmonary tuberculosis reveal that alveolar pneumonia and granuloma cores represent distinct and coexisting immunologic compartments within the same lung. While prior studies have described macrophage heterogeneity in tuberculosis, including foamy macrophages and TREM2-associated macrophage programs, these have largely been examined without spatial resolution or direct comparison of lesion compartments in human tissue. Here, by analyzing adjacent alveolar and granulomatous regions within individual patients, we demonstrate that macrophage states are not randomly distributed but are spatially organized into functionally distinct niches. The alveolar compartment is enriched for $TREM2^{+}$ lipid-laden macrophages, characterized by lipid metabolic reprogramming, reduced T cell infiltration, and increased Mtb-associated RNA and antigen, whereas adjacent granulomas exhibit organized lymphoid architecture and robust antimicrobial programs. These findings define a lesion-specific immune architecture in which macrophage phenotype, bacterial localization, and adaptive immune responses are compartmentalized, identifying the alveolar niche as a permissive environment for Mtb persistence. This framework reframes macrophage heterogeneity in TB as a spatially defined property of the lesion rather than a purely cell-intrinsic program.”

Comment 1.3.“The conclusions are broadly consistent with the data, but several figures remain mainly descriptive rather than strongly quantitative, and additional quantitative analysis and clearer statistical support would strengthen the claims. I do not see major technical flaws in the methodology or analysis, and the methods appear sound and sufficiently detailed for reproduction, but the interpretation feels underdeveloped and the level of novelty is, in my opinion, insufficient to support publication in the present form. Figures 2–5 are largely descriptive and would benefit from more quantitative analysis. In their current form, the images clearly illustrate the spatial patterns the authors want readers to notice, but the conclusions would be strengthened by additional quantification and clearer statistical support. I would therefore encourage the authors to reconsider these figures with appropriate quantification and to make the key biological contrasts more explicit.”

Response: We also added three new supplementary figures corresponding to Figures 2, 3, and 5, providing detailed CosMx protein and IHC quantification. These additions address the reviewers’ concerns and demonstrate the statistical significance of the data.

REVIEWER #2

Comment 2.1. “The suggestion here, is the alveolar pneumonia regions appear to be replete with macrophages may get rid of recognizable Mtb bacilli from the slide, but without efficiently getting rid of the bacillus-derived material such as TB Lam or TB PPD, is that correct?”

Response: We thank the reviewer for this insightful point and agree that the distinction between clearance of intact bacilli and persistence of bacillus-derived material is critical for interpretation. We agree that bacillary components such as LAM or PPD may persist following bacterial killing and that this must be considered when interpreting tissue staining. In our study, we detect both Mtb-associated antigens and Mtb transcripts within alveolar macrophage–rich regions. We note that

RNA-positive cells are less frequent than antigen-positive cells, consistent with the more rapid degradation of bacterial RNA relative to protein or lipid components. Importantly, we do not assume that RNA and antigen signals derive from the same individual cells. Rather, the presence of both signals within the alveolar compartment supports the interpretation that these regions contain Mtb-associated material and are consistent with ongoing or recent bacterial activity, while not excluding the contribution of residual antigen from non-viable organisms. These clarifications have been incorporated into the revised Results section as follows:

"Quantitative spatial analysis demonstrated increased mycobacterial antigen burden in alveolar regions compared to granulomas in PTB lung tissue (Supplementary Figures 17C and 19A). TB-LAM signal was significantly elevated in alveoli (~3–10-fold; $p \leq 0.01$), and TB-PPD staining was also increased (~2–4-fold; $p \leq 0.05$), consistent with enrichment of bacterial components in these regions."

"A critical question was whether foamy macrophages within the alveolar pneumonia serve as the bacterial reservoir in TB pneumonia. Acid-fast microscopy often detects few bacilli in patient biopsies[1], raising the possibility that organisms persist in non-replicating or masked states. To address this, we employed in situ hybridization for Mtb mRNAs. RNAscope for multiple Mtb transcripts (e.g., esxA, fbpB/85B) revealed clusters of bacterial mRNA signals within macrophages in the alveolar pneumonia, even in regions where acid-fast staining was negative (Figure 5D; Supplemental Figure 15)[2]. By contrast, organized granulomas contained only sparse Mtb mRNA puncta, typically appearing as isolated single dots within occasional macrophages. The spatial distribution of Mtb mRNA closely paralleled the pattern of PPD staining (Figure 5C). Quantification of single-cell Mtb mRNA burden demonstrated significantly greater transcript abundance in alveolar pneumonia than in granulomas (Wilcoxon matched-pairs test, $P < 0.001$) (Supplemental Figure 19B)[2]. Although the absolute difference appeared modest on a log scale, paired analysis demonstrated a consistent increase in alveolar regions across all samples. Thus, the greatest burden of Mtb mRNA and antigen localized to the alveolar pneumonia rather than the granuloma cores.

Because bacterial mRNA is highly labile, its detection provides complementary evidence of ongoing or recent bacterial activity. Taken together, these data indicate that Mtb mRNA and Mtb antigen are enriched within macrophages in the alveolar pneumonia. While antigen staining was more widespread, consistent with persistence of bacillary components, the presence of Mtb transcripts within a subset of macrophages supports ongoing or recent bacterial activity in this compartment. We do not assume that Mtb mRNA and antigen signals derive from the same individual cells; rather, their co-localization at the level of tissue regions supports the interpretation that alveolar pneumonia contains abundant Mtb-associated material and is consistent with a compartment permissive for bacterial persistence, while not excluding the contribution of residual antigen from non-viable organisms."

Comment 2.2. "It would be interesting to see how all trans retinoic acid (ATRA) performs, side by side, in the same assays performed in figure 7 (as a positive control)."

Response: We agree that it would be interesting to compare the effect of ATRA vs. vitamin D. While additional experiments are beyond the scope of this revision, we have added the rationale for this in the discussion.

"Other immunometabolic pathways may similarly regulate this macrophage state. For example, all-trans retinoic acid (ATRA) has been reported to induce antimicrobial responses in Mtb–infected macrophages[3], promote autophagy[4], and modulate lipid metabolism[5], suggesting that targeting lipid handling pathways may represent a broader strategy to reprogram permissive macrophage niches."

REVIEWER #3

Major Comments

Comment 3.1. "The study is based on only four patients, all with sputum-negative, biopsy-required early/atypical presentations. This limits generalizability. Please add a supplemental Table 1 with detailed clinical information for each patient and discuss selection bias and generalizability in the Discussion."

Response: We thank the reviewer for this important point. We have added Supplementary Table 1. We have revised the Discussion to include a unified limitation section explicitly addressing biopsy selection bias, disease stage enrichment, and sex-related generalizability.

"A limitation of this study is the selection of biopsy-required cases, which introduces potential bias toward atypical or sputum-negative presentations of pulmonary tuberculosis. These cases likely represent early or non-cavitary disease in which bacilli remain largely confined within alveolar compartments rather than draining into the airways. As such, this cohort may be enriched for alveolar pneumonia lesions and macrophage-dominated immune responses, and may not fully capture the spectrum of advanced cavitary disease. However, the presence of adjacent alveolar and granulomatous regions within the same lung provides a unique opportunity to define lesion-specific immune programs independent of inter-patient variability.

In addition, all patients in this study were male, which may further limit generalizability. Tuberculosis exhibits well-documented sex differences, with adult pulmonary TB occurring more frequently in males and often associated with higher bacterial burden and more severe disease[6]. These differences are thought to reflect both social and biological factors, including sex-specific immune responses. Experimental and clinical studies indicate that sex hormones modulate host immunity, with testosterone generally exerting immunosuppressive effects and estrogen enhancing macrophage activation and antimicrobial responses[7, 8]. While the present study is not designed to assess sex-specific differences in lesion architecture or macrophage phenotypes, future studies including both sexes will be important to determine whether the TREM2^{+} lipid-laden macrophage program and associated alveolar niche differ by sex."

Comment 3.2. "There are no non-TB controls (healthy lung or other inflammatory lung diseases). It is hard to know whether the TREM2^{+} foamy macrophage program is specific to TB or a general response to alveolar lipid accumulation. The authors should either add such controls or clearly tone down claims of a 'TB-specific permissive niche'."

Response: We thank the reviewer and agree that distinguishing phenotype specificity from disease context is essential. To address this, we added frequencies of TREM2^{+} macrophages from the literatures as well as derived from a lung atlas of scRNA-seq data. Together, these findings indicate that the TREM2-associated macrophage program identified in pulmonary tuberculosis represents a conserved disease-associated macrophage state rather than a TB-specific phenotype.

Accordingly, we have revised the manuscript to avoid implying TB specificity. We no longer describe a "TB-specific permissive niche," but instead refer to a "TB-associated alveolar permissive niche." The distinguishing feature in tuberculosis is therefore not the presence of TREM2^{+} macrophages alone, but their spatial organization within the alveolar compartment together with increased Mtb transcripts and antigens, lipid accumulation, and reduced adaptive immune engagement.

These clarifications have been incorporated into the manuscript as follows:

Results: "Across macrophage subsets, TREM2^{+} macrophages comprised approximately 25–30% of the total macrophage compartment when including alveolar, interstitial, SPP1^{+}, and IFN-γ–activated macrophages (Supplementary Figure 4), indicating that this represents a substantial macrophage population." And:

"To determine whether the TREM2^{+} macrophage program identified in pulmonary TB is present in other lung diseases, we analyzed TREM2 expression and TREM2-associated macrophage signature scores across macrophage clusters from the human lung cell atlas (Supplementary

Table 8). The TREM2-associated signatures included the pulmonary TB TREM2⁺ macrophage scRNA-seq signature identified in this study, as well as lists of recurrent genes present in 20+ and 12+ published TREM2⁺ macrophage signatures. Clusters 6, 8, and 11 showed the most consistent enrichment across the three TREM2-associated signatures. When average TREM2 expression was considered together with signature enrichment, a partially overlapping set of TREM2-high and TREM2-signature-enriched macrophage states emerged, including clusters 3, 8, 11, 14, and 17. Disease-to-cluster frequency analysis showed that these macrophage states were present across multiple disease contexts, including sarcoidosis, non-specific interstitial pneumonia, hypersensitivity pneumonitis, COVID-19, and interstitial lung disease, as well as among macrophages from normal lung. Published studies further show that TREM2⁺ macrophages increase in diseased lung relative to control or baseline lung in COPD, NSCLC, pulmonary fibrosis/IPF, and SARS-CoV-2 infection[9, 10, 11, 12] (Supplementary Table 9). Together, these findings indicate that TREM2-associated macrophage programs are not exclusive to TB, but are present at baseline and expanded or redistributed across inflammatory and fibrotic lung disease settings. In our PTB scRNA-seq dataset, TREM2⁺ macrophages comprised approximately 25–30% of the total macrophage population when alveolar, interstitial, SPP1⁺, and IFN-γ–activated macrophage subsets were considered. Thus, TREM2⁺ macrophages represent a substantial macrophage subset in pulmonary TB, but comparison with the human lung atlas indicates that the transcriptional program itself is shared across lung disease contexts."

Discussion: "In our PTB scRNA-seq dataset, TREM2⁺ macrophages comprised approximately 25–30% of the total macrophage population, representing a substantial subset in pulmonary TB. Our mining of the human lung cell atlas and review of the literature revealed that TREM2-associated macrophage programs are also present or enriched across other pulmonary diseases, including chronic obstructive airway disease, fibrotic/interstitial lung disease, viral lung infection, and lung cancer, while remaining detectable at lower levels in normal lung. Thus, our findings do not establish TREM2⁺ foamy macrophages as a TB-specific macrophage phenotype. Rather, the distinguishing feature in pulmonary TB is the spatial organization of this conserved macrophage program within the alveolar airspace and its association with Mtb transcripts and antigens, lipid accumulation, impaired autophagy, and reduced adaptive immune engagement. We therefore refer to this compartment as a TB-associated alveolar permissive niche."

Comment 3.3. "The mechanistic link to TREM2 is correlative. In-vitro experiments rely on IL-4 polarization, and there is no loss-of-function validation (TREM2 knockdown/knockout, blocking antibody, or DAP12 inhibitor) for PDIM/mycolic acid effects or the vitamin D rescue. These experiments are needed to establish causality. I think it would also be necessary to perform TREM2 knockout experiments using immortalized cell lines or TREM2 knockout mice."

Response: We thank the reviewer for this important point and agree that our data establish association rather than direct causality for TREM2 in this context.

Our study integrates spatial, functional, and metabolic analyses to define a macrophage state characterized by TREM2 expression, lipid accumulation, reduced antimicrobial activity, and increased intracellular Mtb survival. We further show that vitamin D–mediated reprogramming reduces TREM2 expression (flow cytometry) and restores antimicrobial function, supporting a link between this macrophage phenotype and bacterial persistence.

We acknowledge that direct loss-of-function experiments (e.g., TREM2 knockout or inhibition) would be required to establish causality. However, such approaches are technically challenging in primary human TREM2⁺ macrophages, and existing models (e.g., murine macrophages or THP-1 cells) do not fully recapitulate human TREM2⁺ macrophage biology.

We have therefore revised the manuscript to avoid causal language and instead frame TREM2 as a marker and component of a macrophage state associated with impaired antimicrobial function and enhanced Mtb survival. These clarifications have been incorporated throughout the revised manuscript.

Comment 3.4. “Bacterial presence in tissue is inferred from RNAscope mRNA and LAM/PPD staining. Given the AFB-negative biopsies, please provide quantitative puncta counts per cell/ROI with statistics and openly discuss the limitations of these readouts (e.g., mRNA half-life, detection of non-viable organisms).”

Response: We thank the reviewer for this important point. We have added quantitative RNAscope puncta counts per cell and per ROI with statistical comparisons between alveolar and granuloma compartments. In addition, we quantified mycobacterial antigen burden using IHC for TB-LAM and TB-PPD, with measurements normalized to nuclear area. We have also expanded the Discussion to clarify the limitations of these readouts, including mRNA stability and antigen persistence independent of bacterial viability.

We have also expanded the Discussion to explicitly address the limitations of these readouts, including the rapid degradation of bacterial mRNA and the potential persistence of antigen from non-viable organisms.

These additions are reflected in the revised Results and Discussion sections.

Comment 3.5. “Some key methodological details are missing or unclear (exact ROI numbers per patient/compartment, multiple-testing correction, batch-effect handling, Jaccard index thresholds). These should be clarified in the Methods or supplement to meet current standards for spatial omics studies.”

Response: These details are now explicitly described in the Methods and Supplementary Information:

- ROI numbers per compartment, highlighted explicitly.
- Multiple-testing correction (Benjamini–Hochberg).
- Batch correction (Harmony integration).
- Jaccard index thresholds and the statistical framework used.

Comment 3.6. “To directly address the highlighted relationship between lipid-droplet accumulation and impaired adaptive immunity, it would be valuable to perform co-culture experiments with TREM2$^+$ foamy macrophages and autologous T cells (before and after lipid reduction by vitamin D) to test whether lowering lipid droplets can restore T cell activation or recruitment.”

Response: We thank the reviewer for this insightful suggestion. We agree that co-culture experiments examining interactions between TREM2$^+$ macrophages and autologous T cells would be informative. However, the use of vitamin D in such co-culture systems would confound interpretation, as vitamin D directly modulates T cell proliferation and cytokine production in addition to its effects on macrophages.

To address this concept more directly, we have revised the Discussion as follows:

“Future studies will be needed to determine whether TREM2$^+$ foamy macrophages differ from other macrophage subsets in antigen presentation and T cell activation capacity, including assessment of MHC class I and class II pathways and whether reprogramming of this permissive macrophage state restores adaptive immune activation.”

Minor Comments

Comment 3.7. “Abstract: state n=4 patients and main methods early, and quantify “sparse T-cell infiltration” where possible.”

Response: We have revised the Abstract as follows: “Here, we integrate spatial transcriptomics, single-cell RNA sequencing, high-resolution imaging, and functional assays of lung biopsies from four patients with pulmonary tuberculosis to directly compare alveolar pneumonia with adjacent granulomas.” We have also replaced qualitative descriptions such as “sparse T-cell infiltration” with a more precise description (“reduced T-cell infiltration”) supported by quantitative analysis in the Results.

Comment 3.8. “Several long sentences in the Introduction (especially lines 60–72) would be easier to read if split.”

Response: We have edited the Introduction throughout to improve clarity and readability, including splitting long sentences and simplifying phrasing where appropriate.

Comment 3.9. “Correct the recurring typo “Mb growth” to “Mtb growth” throughout.”

Response: We have corrected all instances of “Mb growth” to “Mtb growth” throughout the manuscript.

Comment 3.10. “All figures and legends need scale bars, statistical annotations, and quantitative data (e.g., puncta counts, lipid droplet area, CFU) shown as bar graphs rather than representative images only.”

Response: All figures and legends have been revised to include scale bars, statistical annotations, and quantitative data. Representative images are now accompanied by quantitative analyses, including puncta counts, lipid droplet measurements, and CFU-based readouts, with appropriate statistical comparisons and sample sizes indicated. Quantitative analyses are now presented alongside representative images in all relevant figures.

Comment 3.11. “Methods: provide source, purity, solvent controls, endotoxin levels and exact concentrations for PDIM and free mycolic acids, plus full details on Mtb strain, MOI and infection conditions.”

Response: We have expanded the Methods to provide full details on Mtb ligands and infection conditions. Specifically, we now include the source of all ligands (BEI Resources), concentrations used (10 µg/mL), solvent preparation (chloroform:methanol), and inclusion of appropriate vehicle controls. We have also clarified that all lipid preparations were of analytical grade purity and addressed endotoxin considerations. In addition, we provide complete details on Mtb strains, MOI, and infection conditions across experiments.

Comment 3.12. “Standardize terminology (“alveolar pneumonia” vs “alveoli” vs “pneumonia lesions”) and give a quantitative definition of “foamy macrophages” (e.g., PLIN2$^+$ area or Oil Red O positivity).”

Response: We have standardized terminology throughout the manuscript (e.g., “alveolar pneumonia,” “alveoli”) for consistency. We have also introduced a quantitative definition of foamy macrophages based on lipid accumulation, specifically PLIN2$^+$ lipid droplet staining, and incorporated this definition into the Introduction and Methods.

Comment 3.13. “scRNA-seq: report cell numbers per patient before/after filtering, clustering resolution, and top marker genes (ideally in a supplemental table).”

Response: We have expanded the scRNA-seq Methods and Supplementary Information to include detailed reporting of cell numbers per patient before and after filtering, clustering parameters (including resolution), and marker genes for each cluster. These data are now provided in supplementary tables. This definition is now consistently applied throughout the manuscript.

Comment 3.14. “Add a Data Availability statement with GEO accession numbers (or commitment to upload upon acceptance).”

Response: We have added a comprehensive Data Availability section, including GEO accession numbers for all sequencing datasets, as well as links to Zenodo and GitHub repositories containing data and analysis code.

Comment 3.15. “The transmission claim is interesting but speculative; consider adding “potentially” or “may facilitate”.”

Response: We have revised the manuscript to use more cautious language regarding transmission, replacing stronger claims with phrasing such as “may facilitate transmission” and “potentially contribute to transmission” to reflect the speculative nature of this aspect.

Reviewer #3 – Remarks on Code Availability. “The GitHub repository contains relevant scripts for GeoMx, scRNA-seq, CosMx, and text mining analyses, organized into clear modules. It links to GEO and Zenodo data. However, the README provides only a high-level description and lacks detailed installation instructions, dependency lists, or step-by-step running guidelines. The code appears usable for reproducing the main analyses with some effort, but improved documentation would make it a more accessible resource for the community.”

Response: We have updated the GitHub repository to include:

- Installation instructions.
- Dependency lists.
- A step-by-step workflow.
- Example input and output files.

Final Statement

Response: We have addressed all reviewer concerns through substantial revisions that enhance the manuscript’s quantitative rigor, transparency, and conceptual clarity while preserving the central biological insight into a spatially defined, TREM2^{+} macrophage–mediated permissive niche in human TB.

References

1. Peyron P*, et al.* Foamy macrophages from tuberculous patients' granulomas constitute a nutrient-rich reservoir for M. tuberculosis persistence. *PLoS Pathog* **4**, e1000204 (2008).

2. Nargan K*, et al.* Spatial distribution of Mycobacterium tuberculosis mRNA and secreted antigens in acid-fast negative human antemortem and resected tissue. *EBioMedicine* **105**, 105196 (2024).

3. Wheelwright M*, et al.* All-Trans Retinoic Acid-Triggered Antimicrobial Activity against Mycobacterium tuberculosis Is Dependent on NPC2. *J Immunol* (2014).

4. Coleman MM*, et al.* All-trans Retinoic Acid Augments Autophagy during Intracellular Bacterial Infection. *Am J Respir Cell Mol Biol* **59**, 548-556 (2018).

5. Matsuoka H*, et al.* Retinoic acid receptor-related orphan receptor α reduces lipid droplets by upregulating neutral cholesterol ester hydrolase 1 in macrophages. *BMC molecular and cell biology* **21**, 32 (2020).

6. Horton KC, MacPherson P, Houben RM, White RG, Corbett EL. Sex Differences in Tuberculosis Burden and Notifications in Low- and Middle-Income Countries: A Systematic Review and Meta-analysis. *PLoS Med* **13**, e1002119 (2016).

7. Klein SL, Flanagan KL. Sex differences in immune responses. *Nat Rev Immunol* **16**, 626-638 (2016).

8. Neyrolles O, Quintana-Murci L. Sexual inequality in tuberculosis. *PLoS Med* **6**, e1000199 (2009).

9. Byers DE*, et al.* Triggering Receptor Expressed on Myeloid Cells-2 Expression Tracks With M2-Like Macrophage Activity and Disease Severity in COPD. *Chest* **153**, 77-86 (2018).

10. Zhang H*, et al.* Immunosuppressive TREM2(+) macrophages are associated with undesirable prognosis and responses to anti-PD-1 immunotherapy in non-small cell lung cancer. *Cancer Immunol Immunother* **71**, 2511-2522 (2022).

11. Fabre T*, et al.* Identification of a broadly fibrogenic macrophage subset induced by type 3 inflammation. *Sci Immunol* **8**, eadd8945 (2023).

12. Upadhyay AA*, et al.* TREM2(+) and interstitial-like macrophages orchestrate airway inflammation in SARS-CoV-2 infection in rhesus macaques. *Nature communications* **14**, 1914 (2023).

Point-by-Point Responses to Reviewer #1 and Reviewer #3

Manuscript ID: NCOMMS-26-016229B

Response to Reviewer #1

Comment 1

I recommend moving the quantifications of Figures 2, 3, 4, and 5 into the main body of the manuscript. This would help avoid presenting images without accompanying quantitative analysis and would strengthen the presentation of the data within the manuscript.

Response:

We thank the reviewer for this valuable suggestion. In response, we moved the key quantitative analyses from the Supplementary Information into the main manuscript. Quantitative data are now incorporated directly into Figures 2, 3, 4, and 5, allowing the representative images and their corresponding statistical analyses to be presented together. This revision provides immediate quantitative support for the histological and immunofluorescence observations and strengthens the presentation of the data. The Results section and corresponding figure legends were revised accordingly.

Comment 2

I also recommend more thorough annotation and development of several supplementary figures. In particular, Supplementary Figures 1, 2, 9, 10, 11, 12, 14, 15, 16, and 18 appear somewhat underdeveloped and would benefit from additional labeling, explanatory details, and figure annotations to improve clarity and facilitate interpretation by readers.

Response:

We appreciate the reviewer's thoughtful suggestion. We have substantially revised the indicated Supplementary Figures by adding additional labels, annotations, explanatory text, and expanded figure legends to improve clarity and facilitate interpretation. We also incorporated missing scale bars where appropriate, defined all abbreviations within each legend, and included information regarding biological replication, statistical analyses, and error bars. These revisions provide a more complete and reader-friendly Supplementary Information.

Response to Reviewer #3

Comment 1

The revised manuscript is substantially improved. The authors have added useful clinical information, additional quantification, comparison with other lung disease datasets, and a clearer discussion of limitations.

Response:

We sincerely thank the reviewer for the positive assessment of our revised manuscript. We appreciate the recognition that the additional clinical information, expanded quantitative analyses, comparison with other lung disease datasets, and revised discussion have strengthened the manuscript.

Comment 2

My remaining concern is mainly interpretive. The data support a TREM2-positive foamy macrophage state associated with lipid accumulation and enrichment of Mtb transcripts/antigens in alveolar regions. However, without TREM2 loss-of-function, blocking, or pathway-inhibition experiments, the study does not establish that TREM2 itself is causally required for lipid accumulation, impaired autophagy, or enhanced Mtb survival.

Response:

We agree with the reviewer and appreciate this important point. We have revised the manuscript to avoid implying that our data establish a causal role for TREM2. Throughout the manuscript, we now consistently describe our findings as identifying a **spatially organized TREM2-associated permissive macrophage niche** characterized by lipid accumulation, reduced antimicrobial programs, impaired autophagy, and enrichment of *Mycobacterium tuberculosis* transcripts and antigens. We also explicitly acknowledge in the Discussion that the present study did not include TREM2 loss-of-function, receptor-blocking, or pathway-inhibition experiments, and therefore does not establish that TREM2 is causally required for lipid accumulation, impaired autophagy, or enhanced intracellular bacterial survival. These revisions ensure that our conclusions accurately reflect the scope of the data.

Comment 3

I do not request additional experiments at this stage. I recommend that the authors make a final textual revision to moderate causal language throughout the manuscript, especially in the title, abstract, results, figure legends, and discussion. The findings should be framed as identifying a spatially organized TREM2-associated permissive macrophage niche, rather than proving a TREM2-dependent causal pathway.

Response:

We thank the reviewer for this valuable recommendation. In response, we carefully revised the title, Abstract, Results, figure legends, and Discussion to consistently moderate causal language throughout the manuscript. Statements implying that TREM2 directly mediates lipid accumulation, impaired autophagy, or bacterial persistence were replaced with language emphasizing that these findings are **associated with** the TREM2-positive macrophage state observed in our study. We now consistently frame our findings as identifying a **spatially organized TREM2-associated permissive macrophage niche**, rather than establishing a TREM2-dependent causal pathway.

Comment 4

The vitamin D data should also be described cautiously as macrophage reprogramming associated with reduced TREM2 expression, lipid droplets, and bacterial viability, not as definitive evidence of a TREM2-specific rescue mechanism.

Response:

We agree and have revised the manuscript accordingly. The Results and Discussion now describe 1,25-dihydroxyvitamin D_3 treatment as inducing macrophage reprogramming that is **associated with** reduced TREM2 expression, decreased lipid droplet accumulation, enhanced autophagy, and reduced intracellular *Mycobacterium tuberculosis* viability. We no longer interpret these findings as demonstrating a TREM2-specific rescue mechanism. Instead, we present them as evidence that the permissive macrophage phenotype is pharmacologically modifiable while acknowledging that additional mechanistic studies will be required to define the specific contribution of TREM2 signaling.

Comment 5

I also suggest that the authors ensure the figure legends and statistical descriptions clearly distinguish donor-level biological replication from ROI- or image-level measurements.

Response:

We thank the reviewer for this important suggestion. We have revised the figure legends and Methods to clearly distinguish donor-level biological replication from ROI- and image-level measurements throughout the manuscript. The revised legends now specify the biological unit analyzed, the number of independent donors, the nature of the quantified measurements, the statistical tests performed, and the definition of the error bars. These revisions improve transparency and ensure compliance with the reporting standards of *Nature Communications*.
