# Supplementary material for: Identification of a spatially organized TREM2-associated permissive macrophage niche in human pulmonary tuberculosis alveolar pneumonia
Source: Nat Commun. 2026 Aug 21;17:10817. doi: 10.1038/s41467-026-76814-8 (PMC13669344; doi:10.1038/s41467-026-76814-8)
Supplement: Supplementary file 3 [file 41467_2026_76814_MOESM1_ESM.pdf]

Supplementary Information

Identification of a spatially organized TREM2-associated permissive macrophage niche in human pulmonary tuberculosis alveolar pneumonia

Rosane M. B. Teles[1], Chaouki Benabdessalem[2], Jonathan Perrie[3], Cenfu Wei[4], Julie West[1], Bruno J. de Andrade Silva[1], Priscila R. Andrade[1], Lilah A. Mansky[1], Prajan Divakar[5], Linda Fischbacher[6], Karen Lam[1], Feiyang Ma[1], Yiqian Gu[3], Kimia Rategh[1], Madeline Brown[1], Aparna Pillai[1], Kapongo Lumamba[7], Threnesan Naidoo[7,8], Samuel M. French[9], Emna Romdhane[10], Mohamed-Ridha Barbouche[2,19], Eynav Klechevsky[11], Marco Colonna[11], Adrie J. C. Steyn[7,8,12], Steven J. Bensinger[13,14], Daniel L. Barber[15], Soumaya Rammeh[10], Parambir S. Dulai[4], Bryan D. Bryson[16], Matteo Pellegrini[3], John T. Belisle[6], Barry R. Bloom[17,18], and Robert L. Modlin[1,13]

This Supplementary Information file contains:

- Supplementary Figures **1–23** and their corresponding figure legends.
- Supplementary Tables **1–7**

The following files are provided separately:

- Supplementary Data files **1–8** (Excel, .xlsx)
- Source Data file (*Excel, .xlsx*)

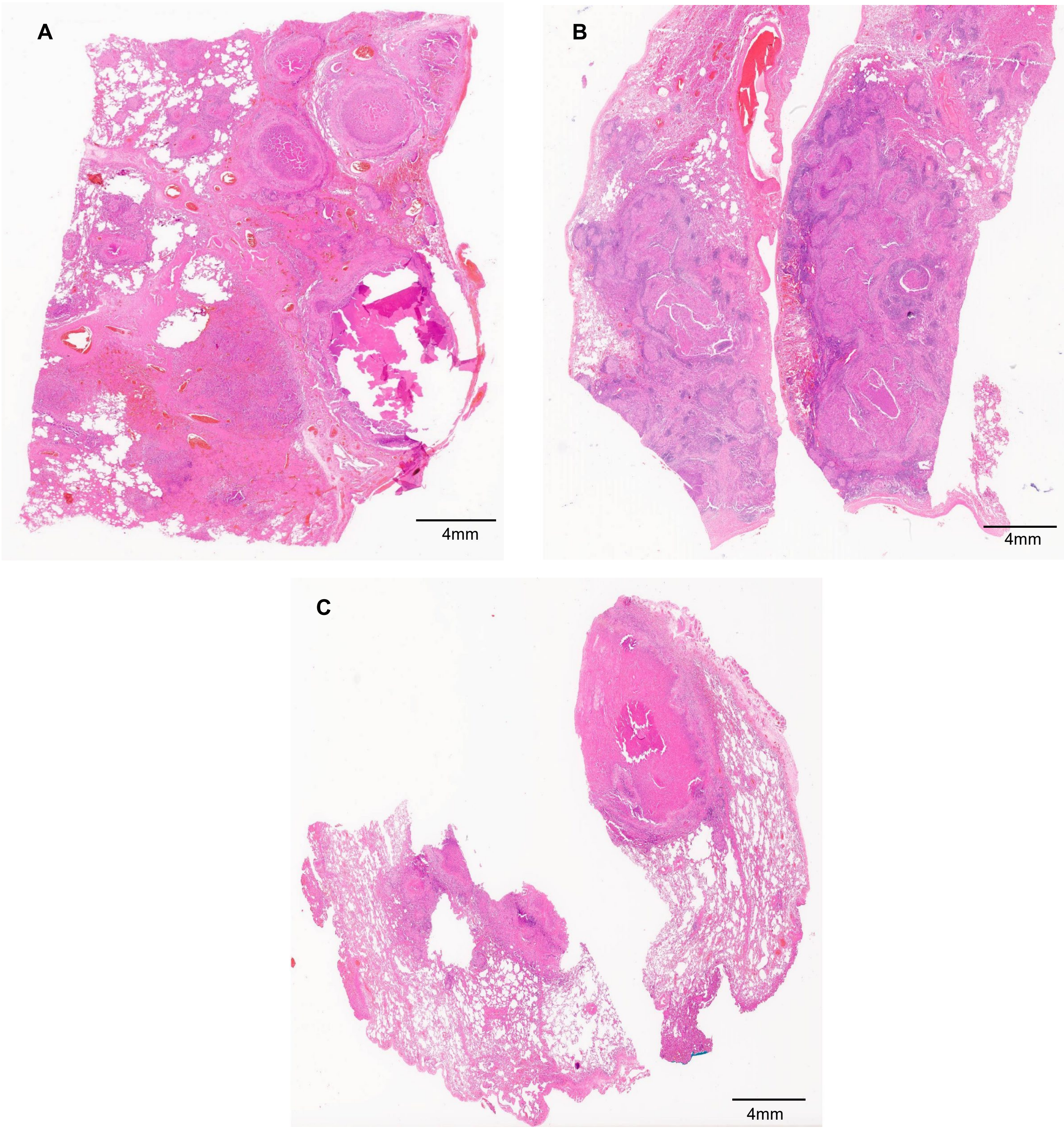

Supplementary Figure 1. Histopathological features of pulmonary tuberculosis specimens used for GeoMx spatial transcriptomic profiling. Representative H&E-stained sections from the three additional PTB donors (PTB 22.1, PTB 22.2, and PTB 22.3) used for GeoMx spatial transcriptomic profiling. PTB 21.1 is presented in Figure 1. Well-organized granulomas are surrounded by areas of alveolar pneumonia containing numerous foamy macrophages occupying alveolar spaces. These specimens were used for selection of regions of interest (ROIs) representing granuloma core, granuloma mantle, and adjacent alveolar tissue for spatial transcriptomic analysis. Representative images are shown from four independent PTB donors (n = 4).

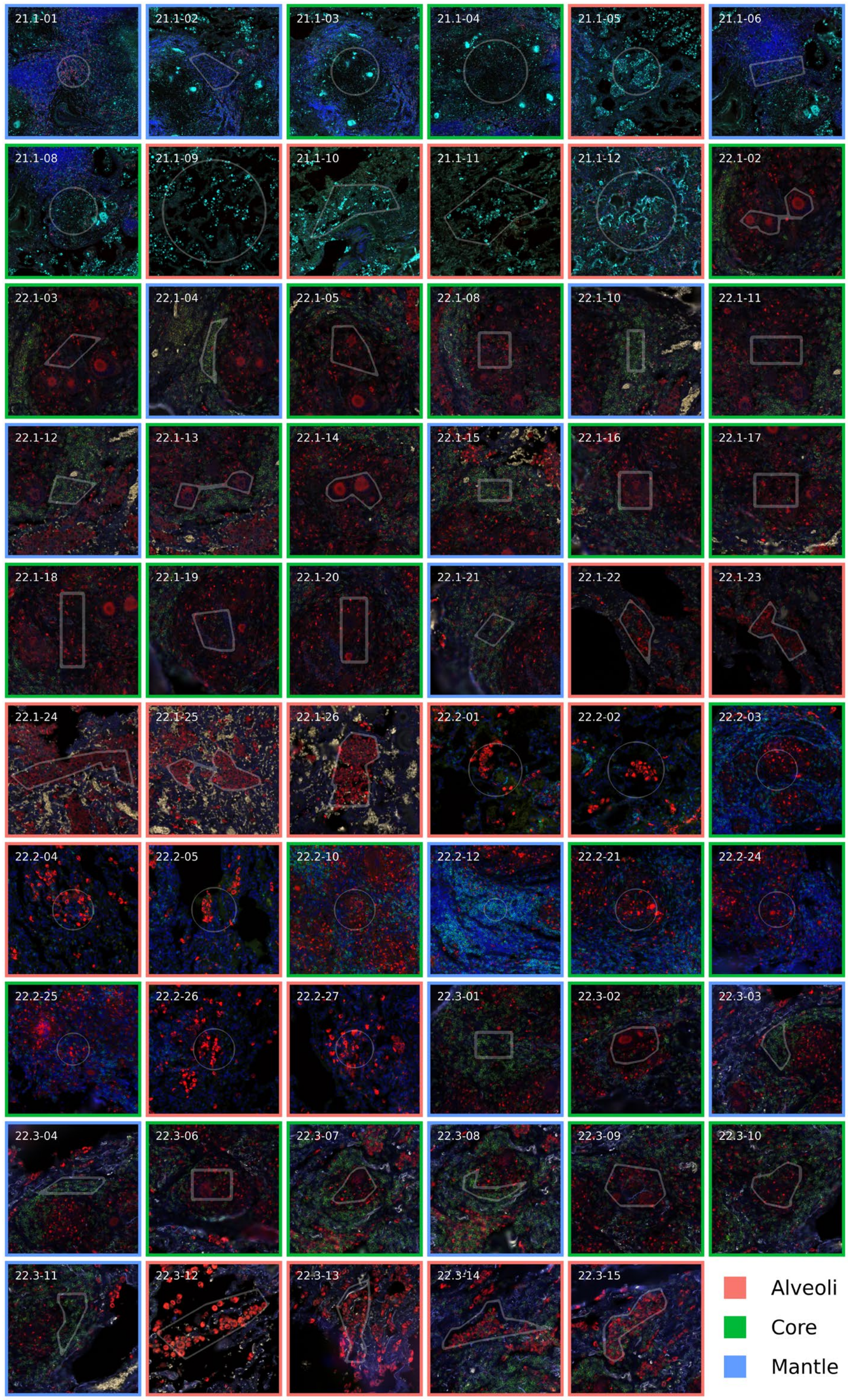

Supplementary Figure 2. Selection of regions of interest (ROIs) for GeoMx Digital Spatial Profiling of pulmonary tuberculosis lesions. Representative tissue sections from four independent pulmonary tuberculosis (PTB) donors (PTB 21.1a, PTB 22.1, PTB 22.2, and PTB 22.3) used for GeoMx spatial transcriptomic analysis. ROIs were selected to capture the major pathological compartments of the lesion, including granuloma core, granuloma mantle, and surrounding alveolar pneumonia containing foamy macrophages. ROI selection was performed on serial immunofluorescence-stained sections to ensure accurate anatomical localization while preserving spatial relationships between granulomatous and non-granulomatous tissue. Multiple ROIs were analyzed from each donor to characterize spatial transcriptional heterogeneity across PTB lesions.

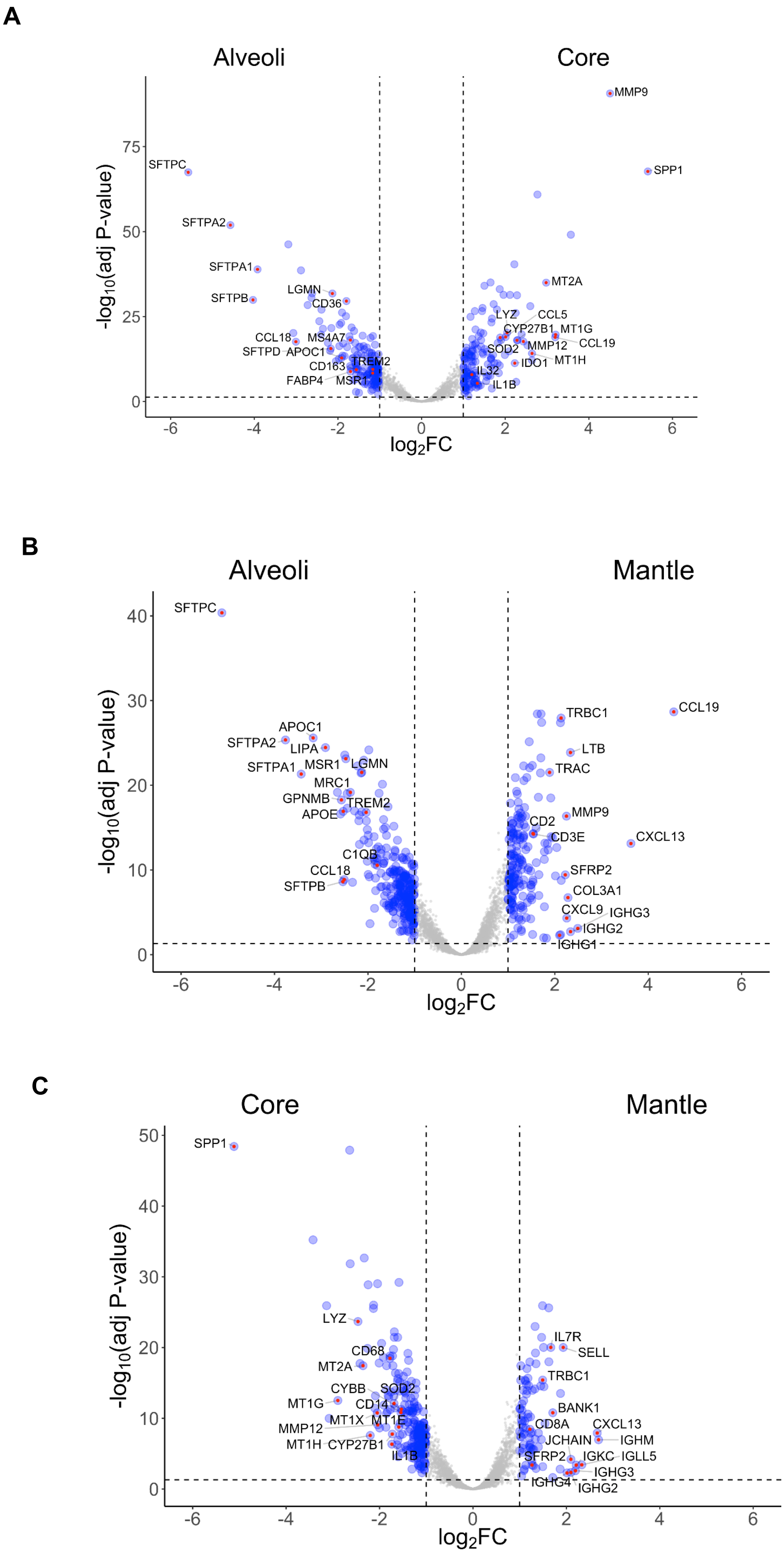

Supplementary Figure 3. GeoMx analysis of pulmonary TB specimens. Volcano plots showing log2FC and –log10(padj) for expression of 202 genes in alveoli vs. core (A), alveoli vs. mantle (B) and core vs. mantle (C).

A

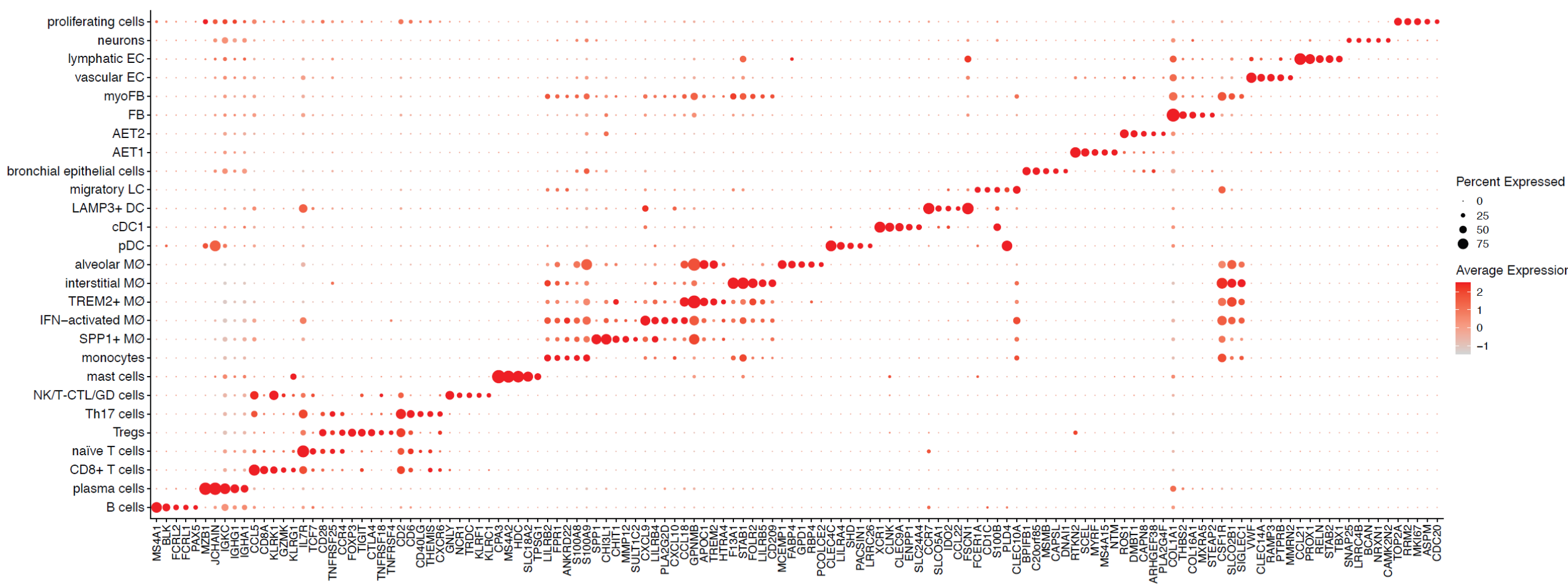

B

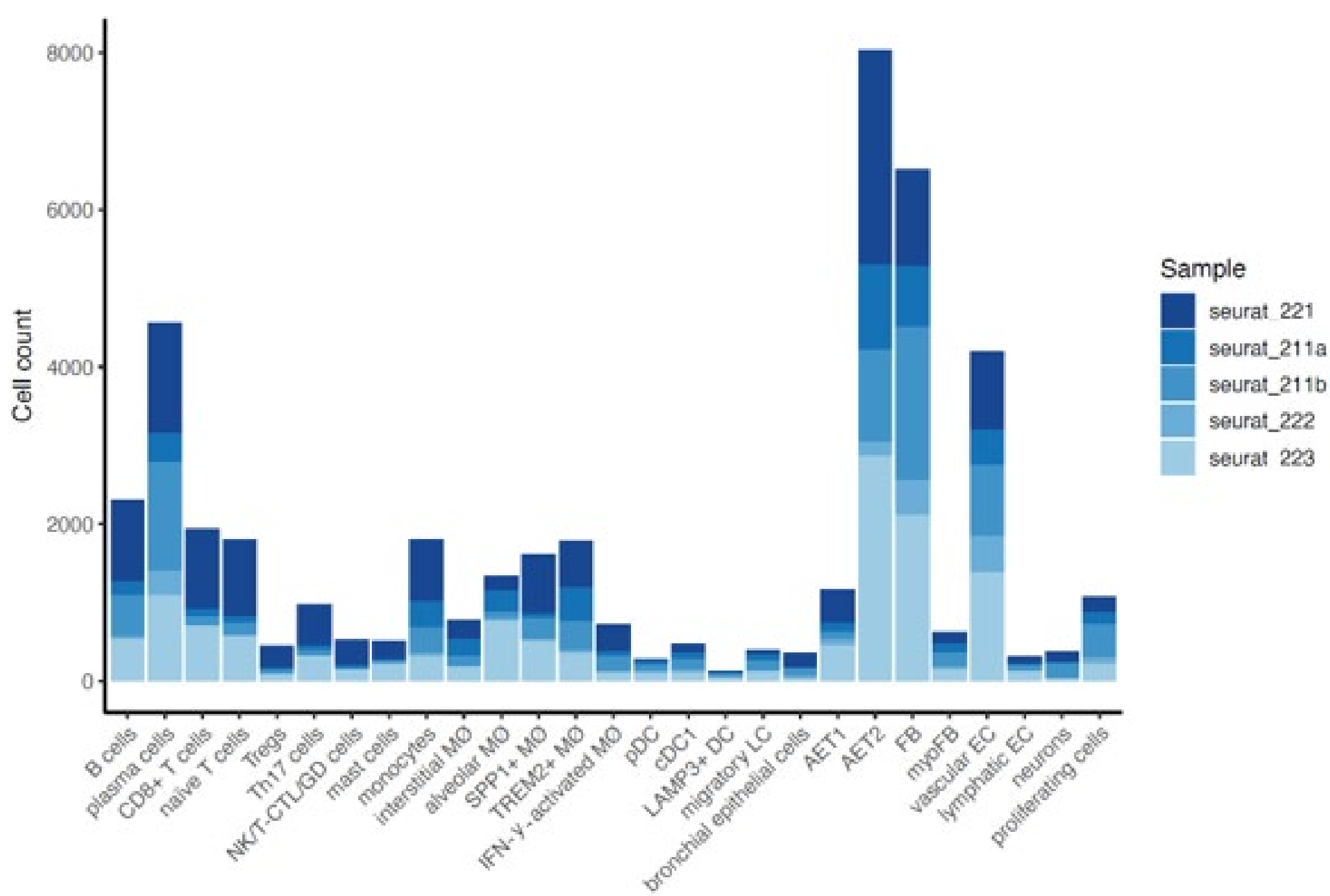

Supplementary Figure 4. Cell cluster definitions for PTB scRNA-seq data. The scRNA-seq data from PTB samples were clustered into major cell types, and then the myeloid and T cell clusters were further subclustered. (A) Top five signature genes in each cluster. (B) Number of cells in each cluster by PTB sample number.

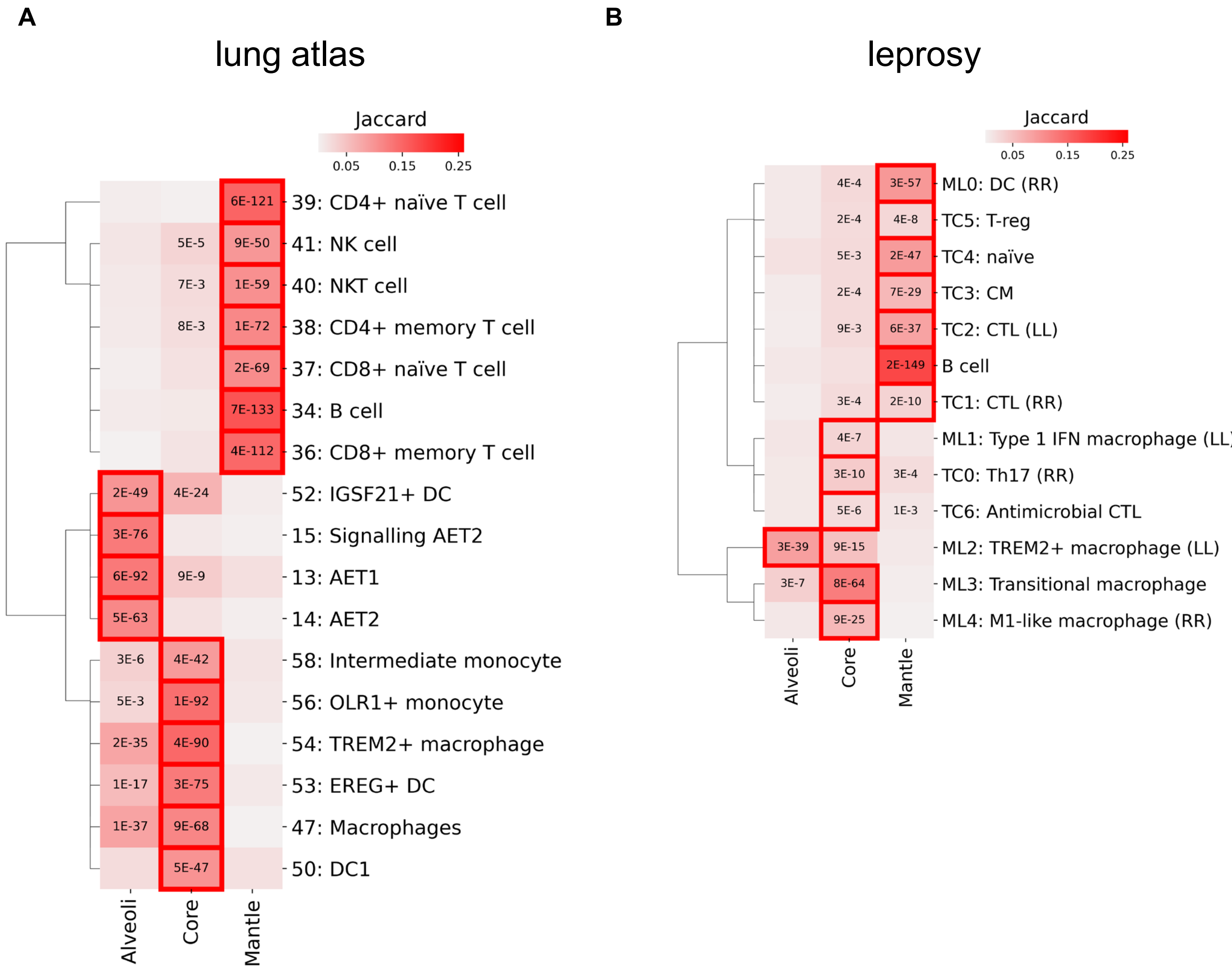

Supplementary Figure 5. Integration of GeoMx pulmonary TB (PTB) spatial profiling data with scRNA-seq atlases. (A) Jaccard overlap of GeoMx PTB spatial profiling data with the human lung atlas scRNA-seq data. (B) Jaccard overlap of GeoMx PTB spatial profiling data with the leprosy scRNA-seq data. Hypergeometric distribution analysis was used for statistical analysis. The p value of the jaccard overlap is shown in each box if significant. For each row (cell type) the lowest p value is highlighted with a red box.

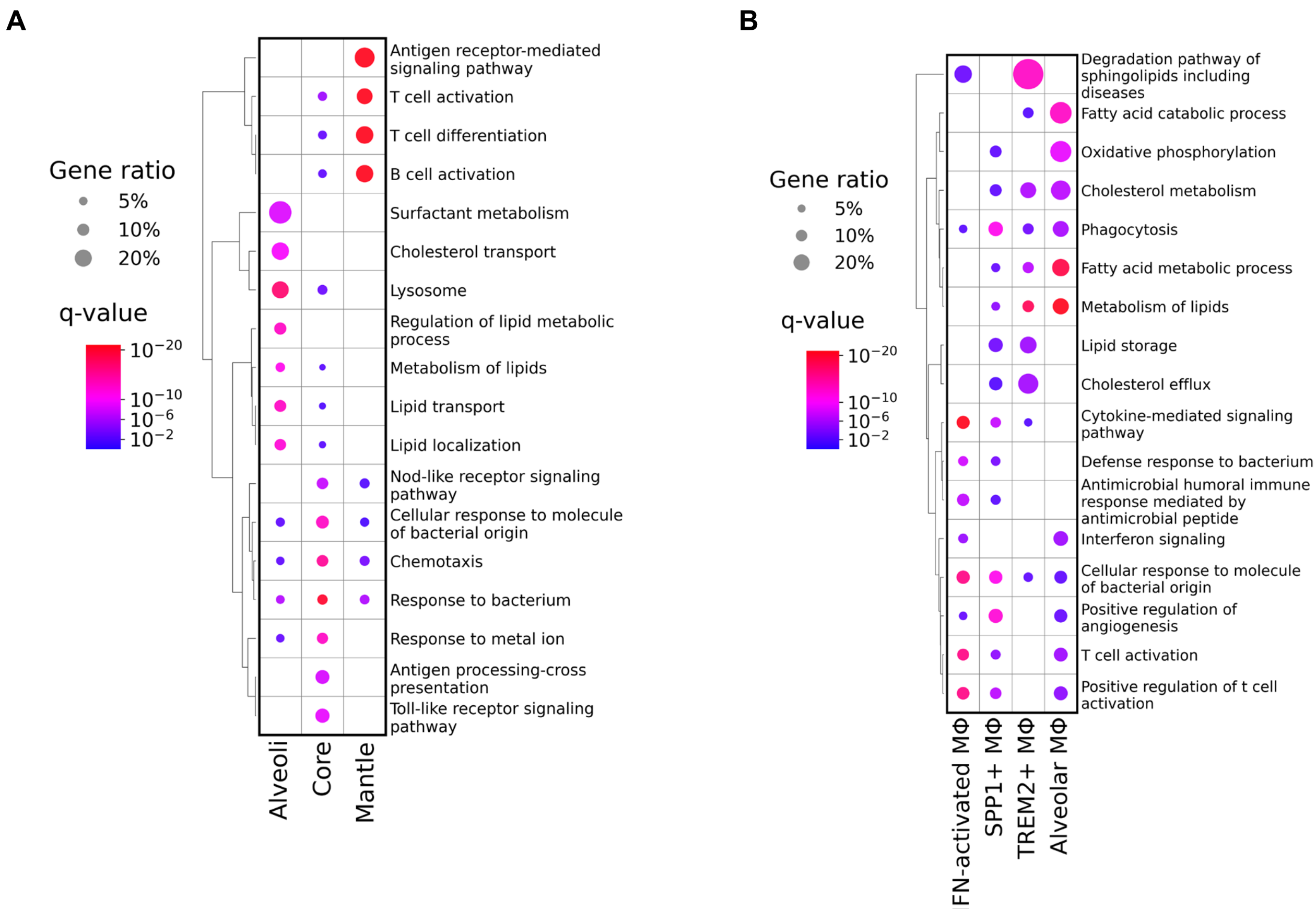

Supplementary Figure 6. Functional pathway analysis of the GeoMx spatial profiling data and scRNA-seq data from human pulmonary TB (PTB). (A) Metascape analysis of GeoMx spatial profiling differential expressed genes (DGEs) from PTB to identify functional pathways. (B) Metascape analysis of scRNA-seq signatures from PTB to identify functional pathways.

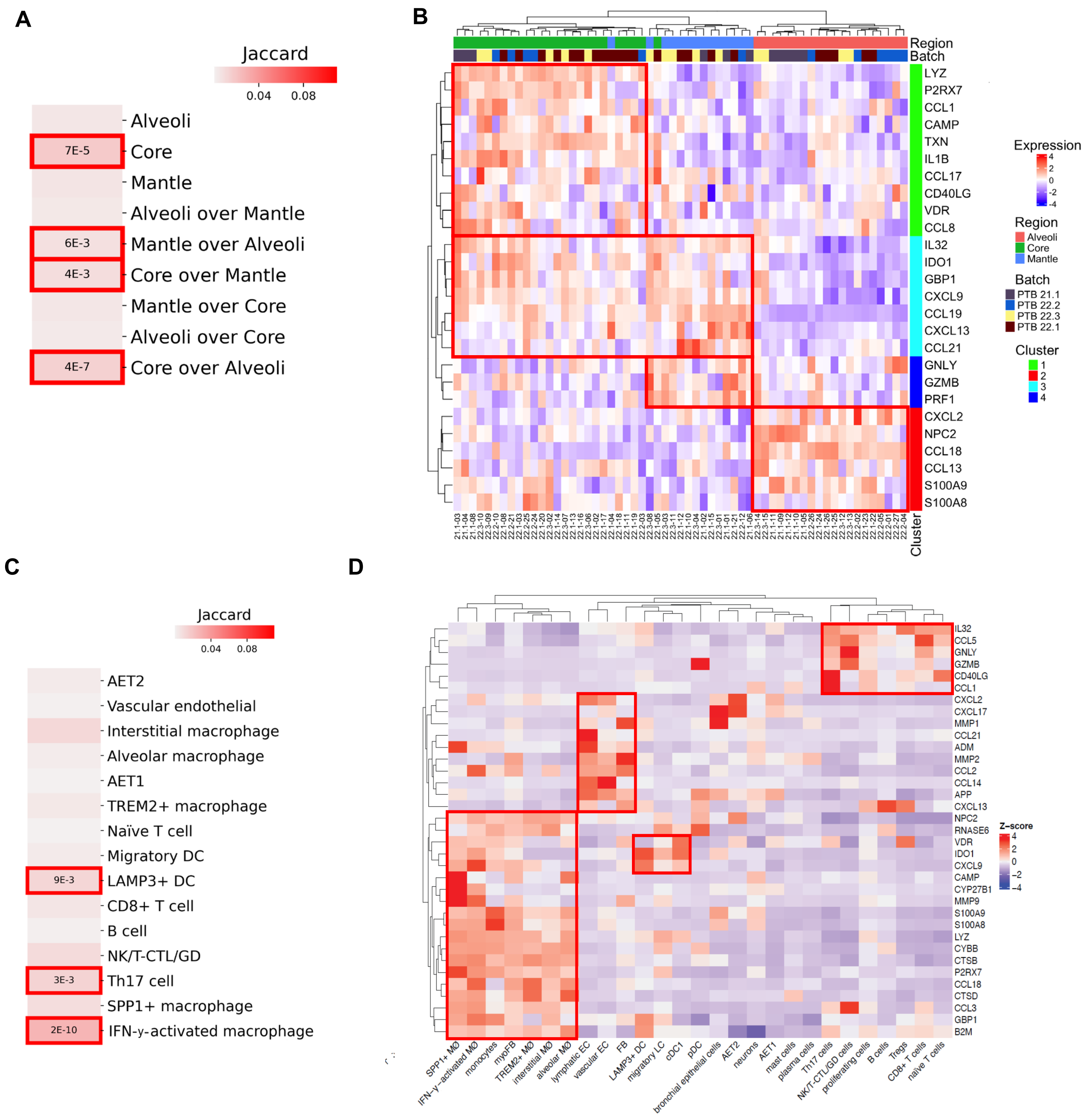

Supplementary Figure 7. Antimicrobial genes signature in PTB samples. (A) Antimicrobial genes expressed in alveoli (PTB A), core (PTB C) and mantle (PTB M) from PTB GeoMx data by Jaccard index and hypergeometic distribution analysis. The p value of the jaccard overlap is shown in each box if significant. (B) Heatmap of antimicrobial gene expression in alveoli, core and mantle form PTB GeoMx data. (C) Antimicrobial genes expressed by the PTB cell types from scRNA-seq data by Jaccard index and hypergeometric distribution analysis. The p value of the jaccard overlap is shown in each box if significant. (D) Heatmap of antimicrobial genes expressed by the PTB cell types from scRNA-seq data.

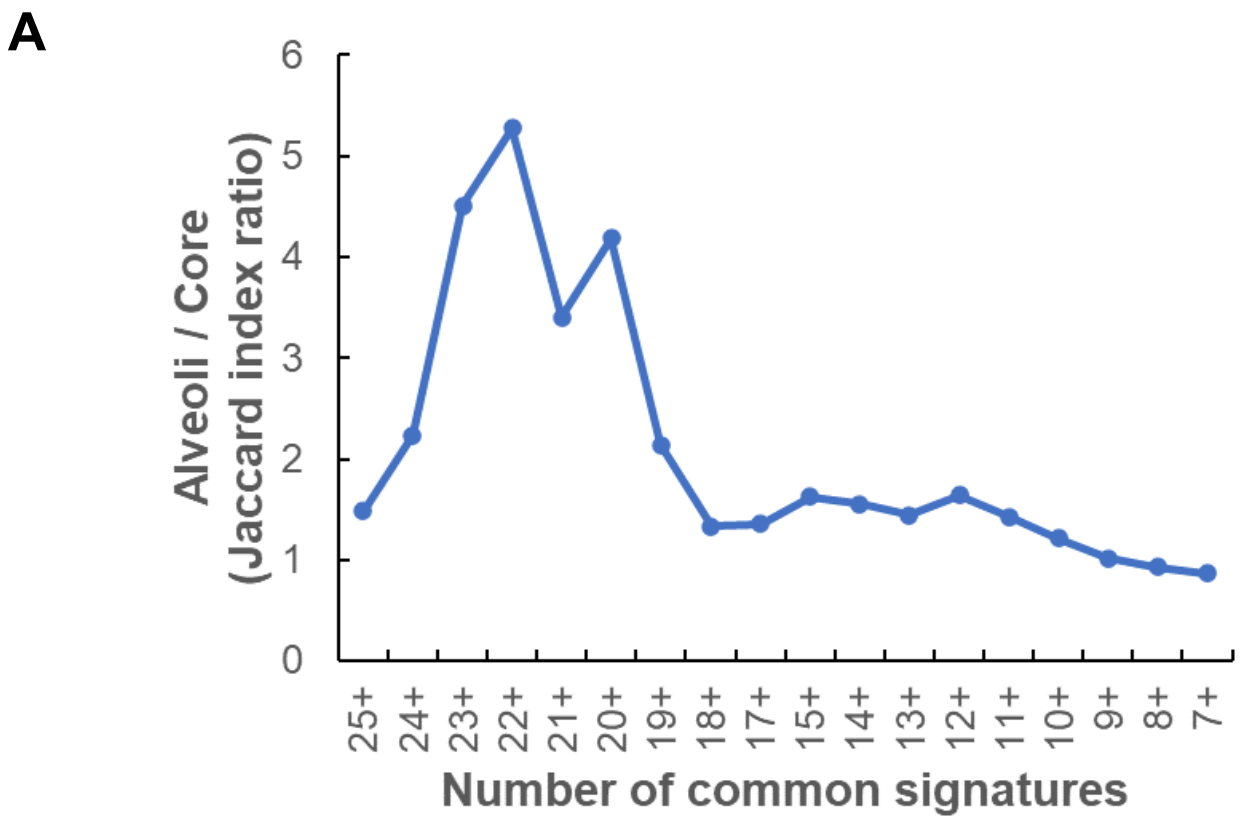

B

Gene	Frequency
TREM2	31
APOE	29
CTSB	27
MS4A6E	25
C1QC	24
C1QB	24
GRN	23
LGMN	23
CTSD	23
C1QA	23
FTL	22
NPC2	22

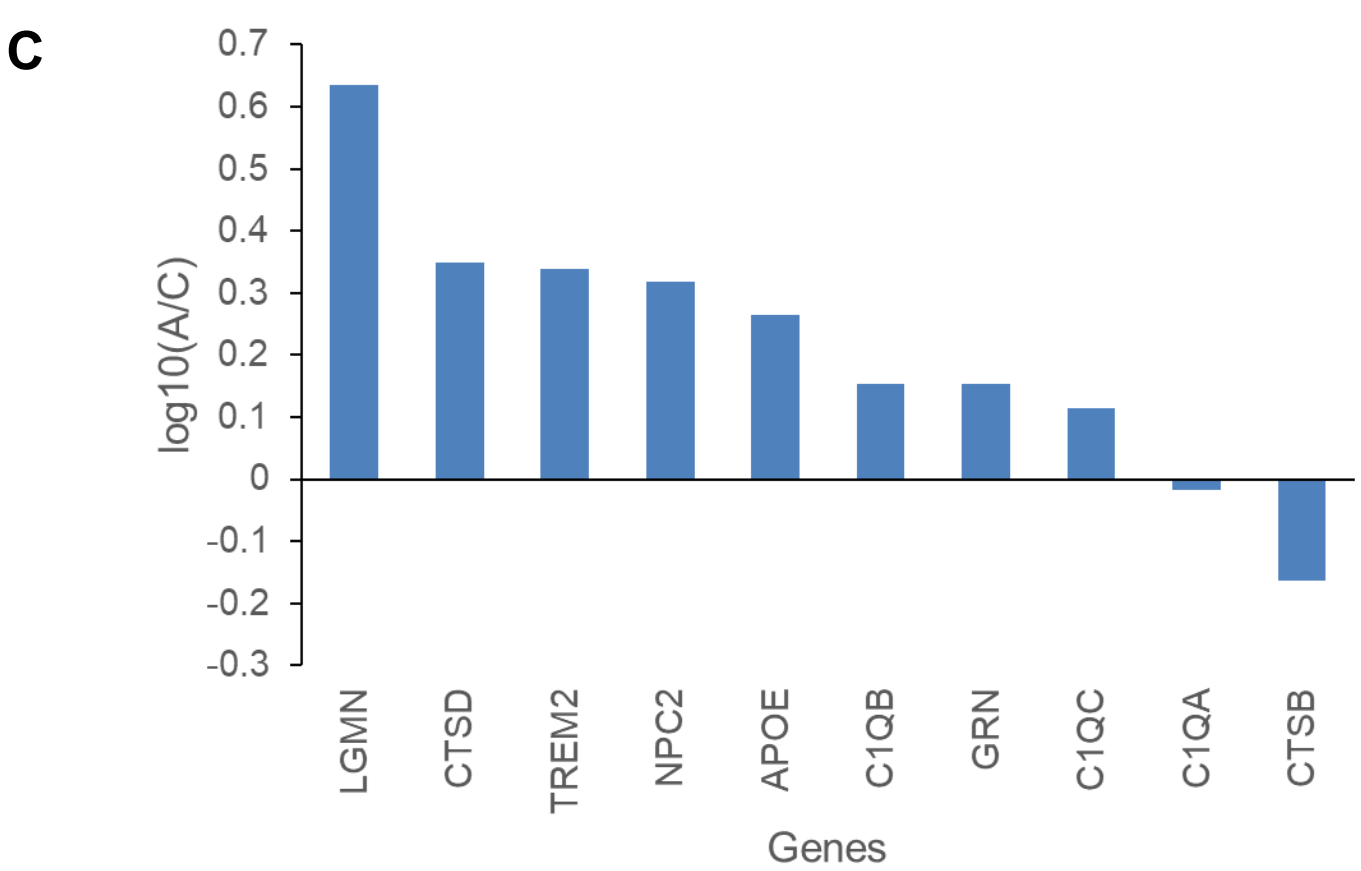

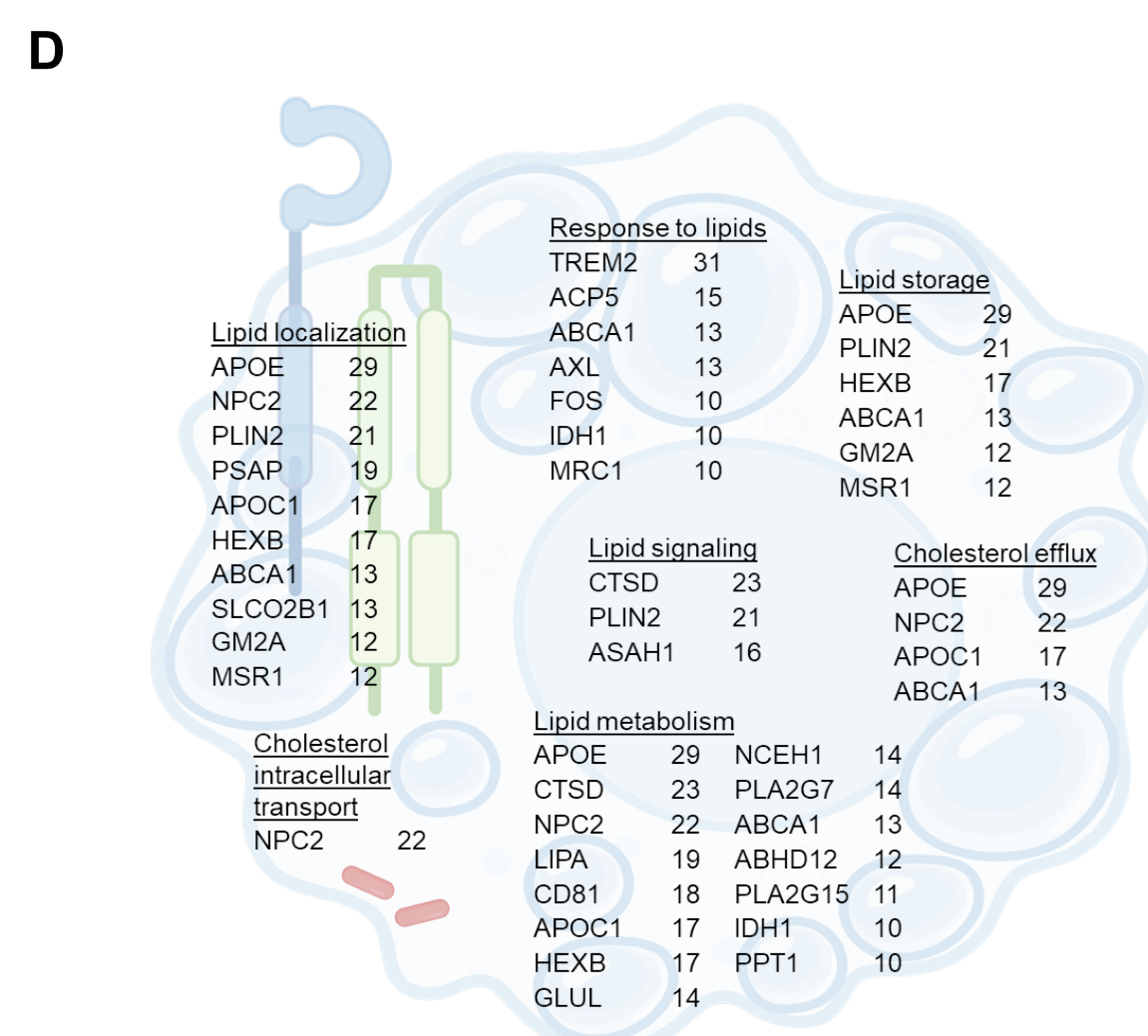

Supplementary Figure 8. Expression of alveoli DGEs in TREM2$^+$ macrophages. (A) The frequency of individual genes within 34 TREM2$^+$ macrophage signatures from 21 publications was calculated and plotted vs. the Jaccard index ratios of these genes in the alveoli/core GeoMX spatial profiling pulmonary TB data. (B) The twelve genes found in 22 or more published TREM2$^+$ macrophage signatures are shown with the frequency of that gene in the signatures. (C) The gene expression ratio (log10, alveoli/core) for genes found in 22 or more TREM2 macrophage signatures. MS4A6E and FTL were not detected in the pulmonary TB GeoMx spatial profiling data. (D) TREM2$^+$ macrophages show enriched expression of genes involved in lipid metabolism, including lipid localization, signaling, response, and cholesterol transport/efflux.

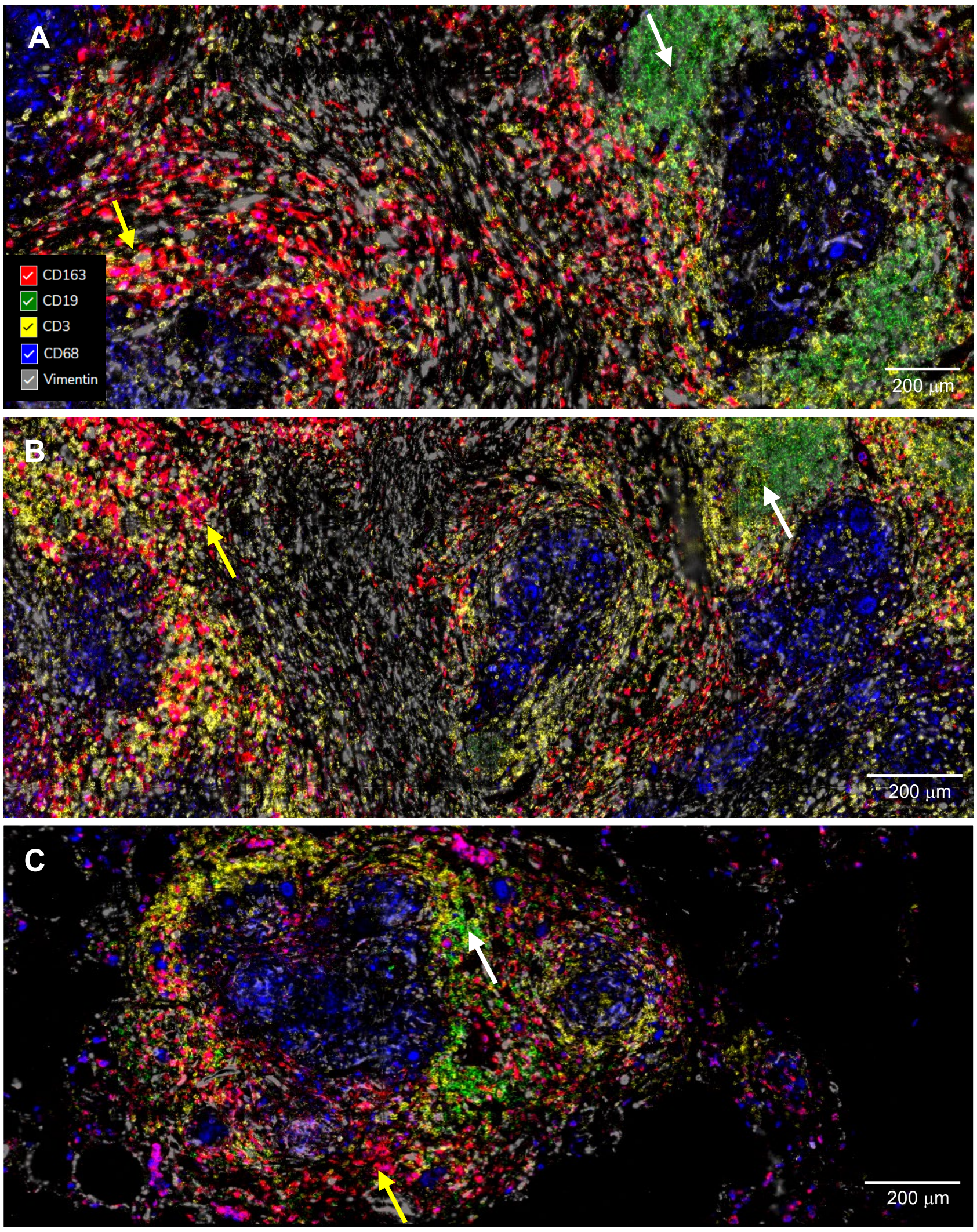

Supplementary Figure 9. CosMx spatial protein profiling demonstrates the organization of immune cell populations within pulmonary tuberculosis lesions. Representative CosMx Spatial Molecular Imager protein images from four independent PTB donors showing the architecture of granulomatous inflammation. (A,B) Distinct granulomas in PTB 22.2 and (C) PTB 22.1 are surrounded by areas of alveolar pneumonia (yellow arrows) enriched in CD163^{+} macrophages. Dense aggregates of CD19^{+} B cells (white arrows) are observed adjacent to granulomas. CD68^{+} macrophages are concentrated within granuloma cores, whereas CD3^{+} T cells are enriched within granuloma mantles. Marker colors are CD163 (red), CD19 (green), CD3 (yellow), CD68 (blue), and vimentin (gray). Representative images are shown from four independent PTB donors (n = 4). Scale bars, 200 µm.

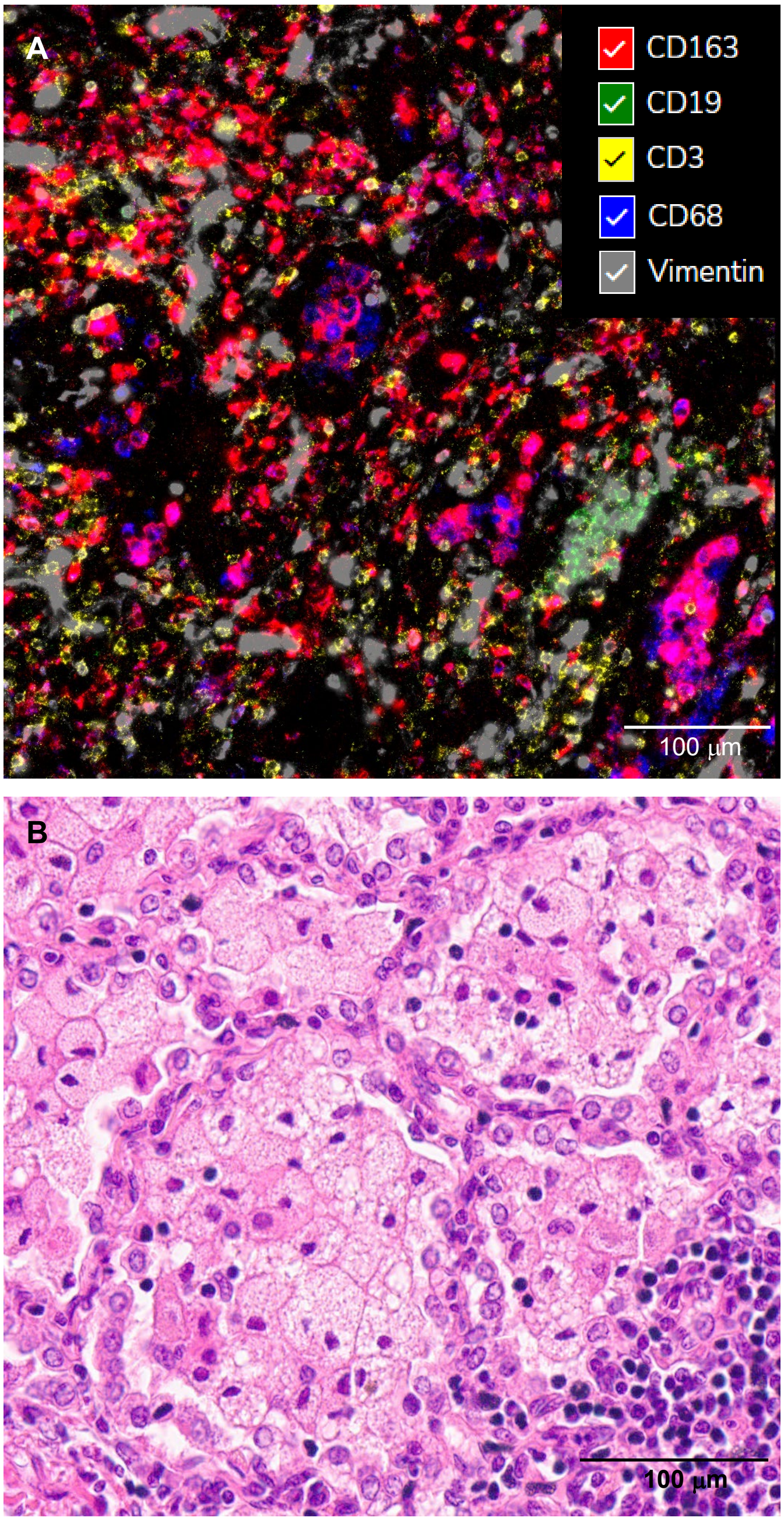

Supplementary Figure 10. Alveolar foamy macrophages identified by CosMx spatial protein profiling. (A) Representative CosMx image demonstrating accumulation of CD163^{+} foamy macrophages (red) within alveolar spaces adjacent to granulomatous inflammation. Alveolar architecture is delineated by vimentin staining (gray), whereas CD68 (blue), CD3 (yellow), and CD19 (green) identify additional immune cell populations. (B) Corresponding H&E-stained serial section confirming the presence of foamy macrophages occupying alveolar spaces in the PTB 22.2 specimen. Representative images are shown from four independent PTB donors (n = 4). Scale bars, 100 µm.

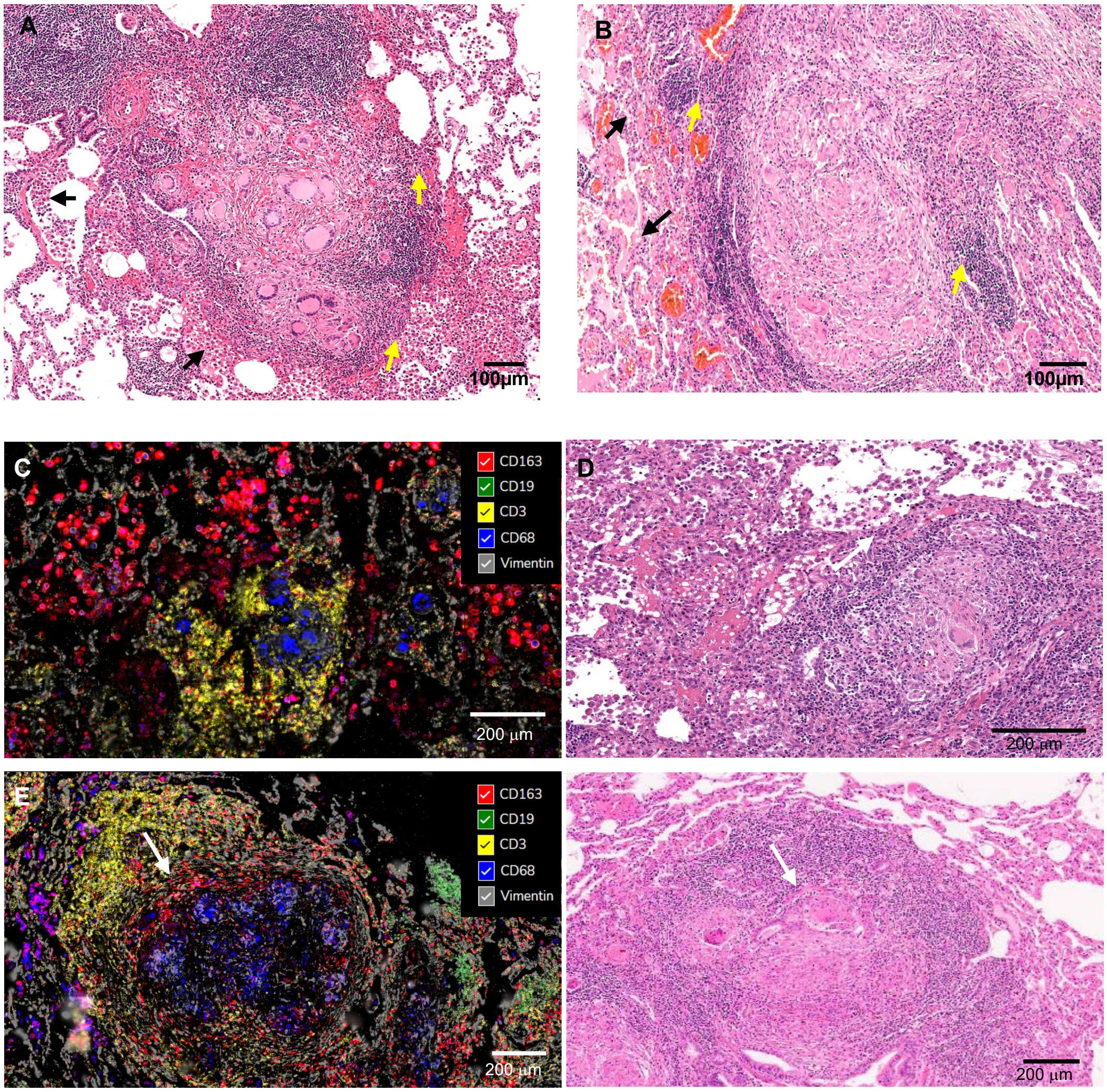

Supplementary Figure 11. Histological organization of granulomas and adjacent alveolar pneumonia in pulmonary tuberculosis. Representative histological and multiplex immunofluorescence images illustrating the spatial relationship between granulomatous inflammation and surrounding alveolar tissue. (A,B) H&E-stained sections showing foamy macrophages filling alveolar spaces (black arrows) adjacent to granuloma mantles (yellow arrows) in PTB 21.1 and PTB 22.2. (C,D) Organized granulomas containing multinucleated Langhans giant cells within the granuloma core surrounded by lymphocyte-rich mantle regions. Dense aggregates of T and B lymphocytes are present adjacent to granulomas. (E,F) CosMx immunofluorescence and corresponding H&E section demonstrating $CD163^{+}$ macrophages located at the interface between granulomas and inflamed alveolar tissue. Marker colors are CD163 (red), CD19 (green), CD3 (yellow), CD68 (blue), and vimentin (gray). Representative images are shown from four independent PTB donors (n = 4). Scale bars, 100 µm (A,B) and 200 µm (C–F).

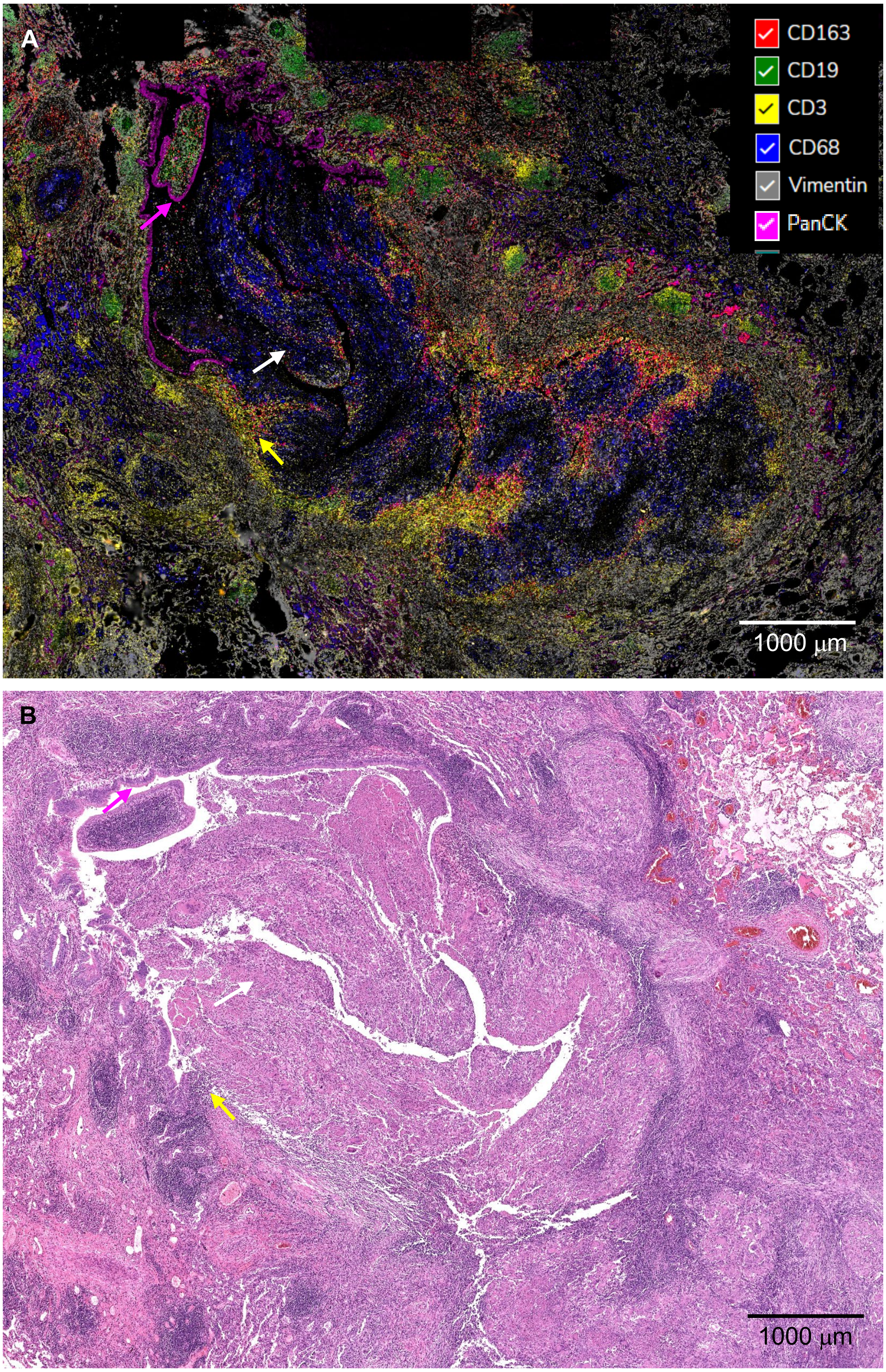

Supplementary Figure 12. Granulomatous inflammation extending into the bronchial wall in pulmonary tuberculosis. Representative CosMx immunofluorescence image (A) and corresponding H&E-stained section (B) from PTB 22.2 demonstrating granulomatous inflammation associated with bronchial obstruction. Pan-cytokeratin (PanCK, purple) identifies the bronchial epithelium, whereas CD163 (red), CD19 (green), CD3 (yellow), CD68 (blue), and vimentin (gray) identify immune cell populations. The bronchial epithelium (pink arrows), T-cell-rich inflammatory infiltrates (yellow arrows), and areas of necrosis (white arrows) are indicated. Representative images are shown from four independent PTB donors (n = 4). Scale bars, 1000 µm.

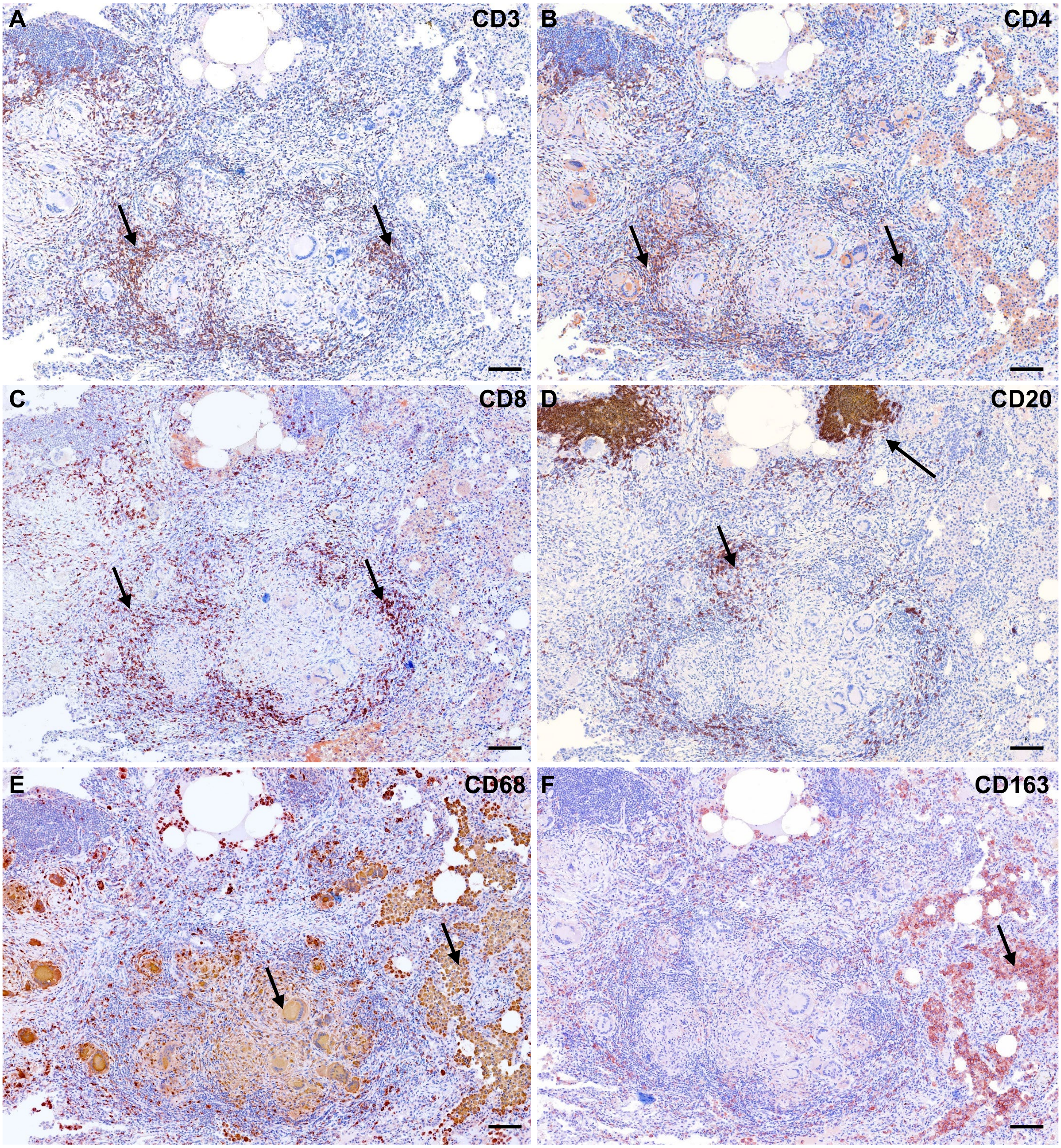

Supplementary Figure 13. Immunohistochemical characterization of immune cell populations in pulmonary tuberculosis lesions demonstrates spatial compartmentalization of lymphoid and myeloid cells. Representative immunohistochemical staining was performed on serial sections from four independent pulmonary tuberculosis (PTB) donors (n = 4) to define the distribution of major immune cell populations within granulomatous lesions and adjacent alveolar pneumonia. (A–C) $CD3^+$, $CD4^+$, and $CD8^+$ T cells are predominantly localized within the granuloma mantle surrounding the necrotic core, consistent with the organization of adaptive immune responses in established granulomas. (D) $CD20^+$ B cells are enriched within perigranulomatous lymphoid aggregates and are also present within the granuloma mantle. (E) $CD68^+$ macrophages are abundant within the granuloma core and are additionally detected in alveolar regions surrounding granulomatous lesions. (F) $CD163^+$ macrophages are predominantly localized to alveolar pneumonia, where they are associated with foamy macrophage-rich areas adjacent to granulomas. Black arrows indicate representative positively stained cells. Representative images are shown from four independent PTB donors. Scale bars, 100 μm.

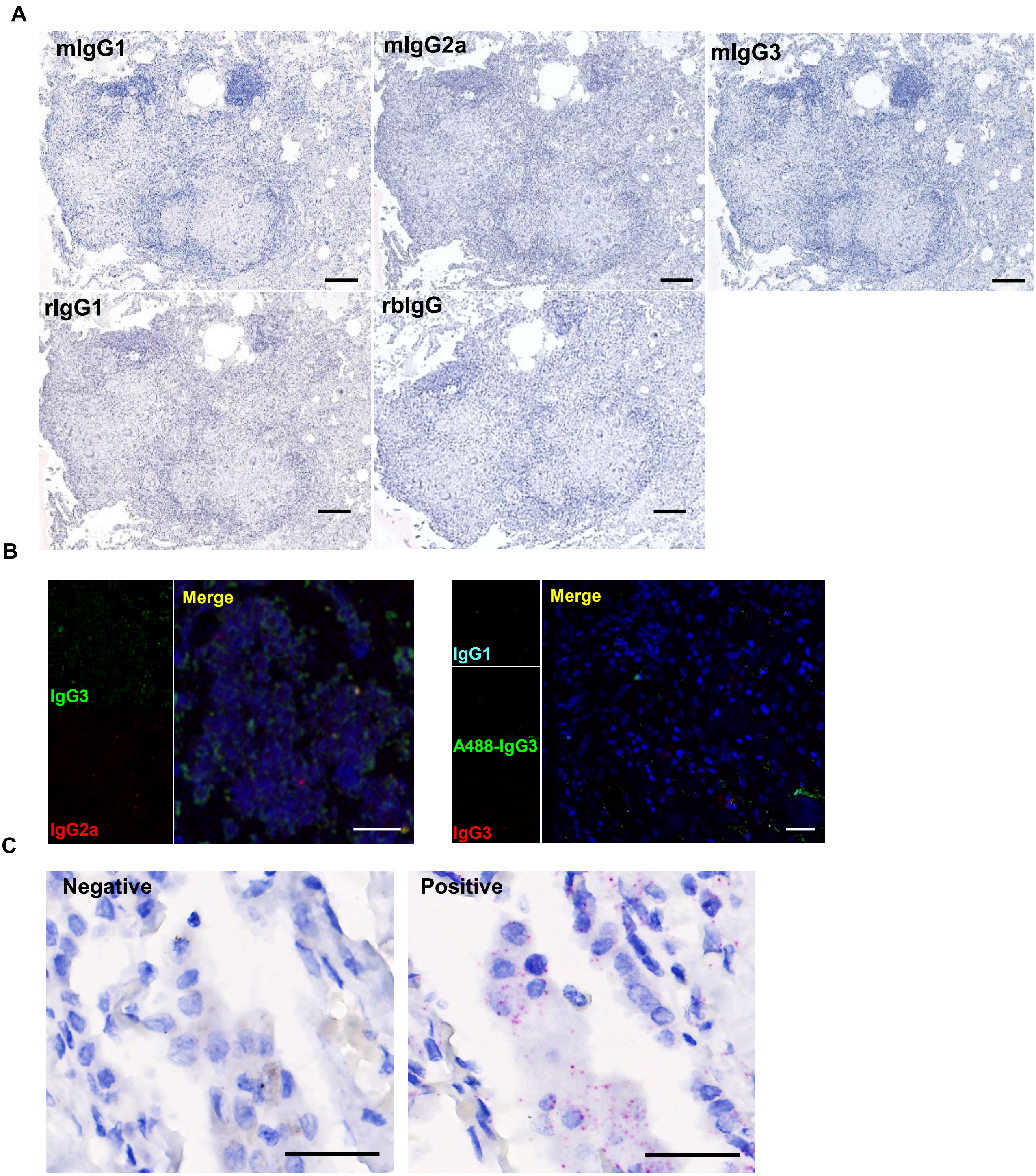

Supplementary Figure 14. (A) Negative controls for immunohistochemistry, immunofluorescence, and RNAscope analyses. (A) Representative isotype control staining for all antibodies used in this study, including mouse IgG1, mouse IgG2a, mouse IgG3, rabbit IgG, and rat IgG1 controls corresponding to their respective primary antibodies. (B) Representative immunofluorescence isotype controls demonstrating absence of nonspecific fluorescence under the same acquisition conditions used for experimental samples. (C) Representative RNAscope positive and negative control probes confirming RNA integrity and assay specificity in alveolar foamy macrophages. Representative images are shown from four independent PTB donors (n = 4). Scale bars, 100 μm (A) and 20 μm (B,C).

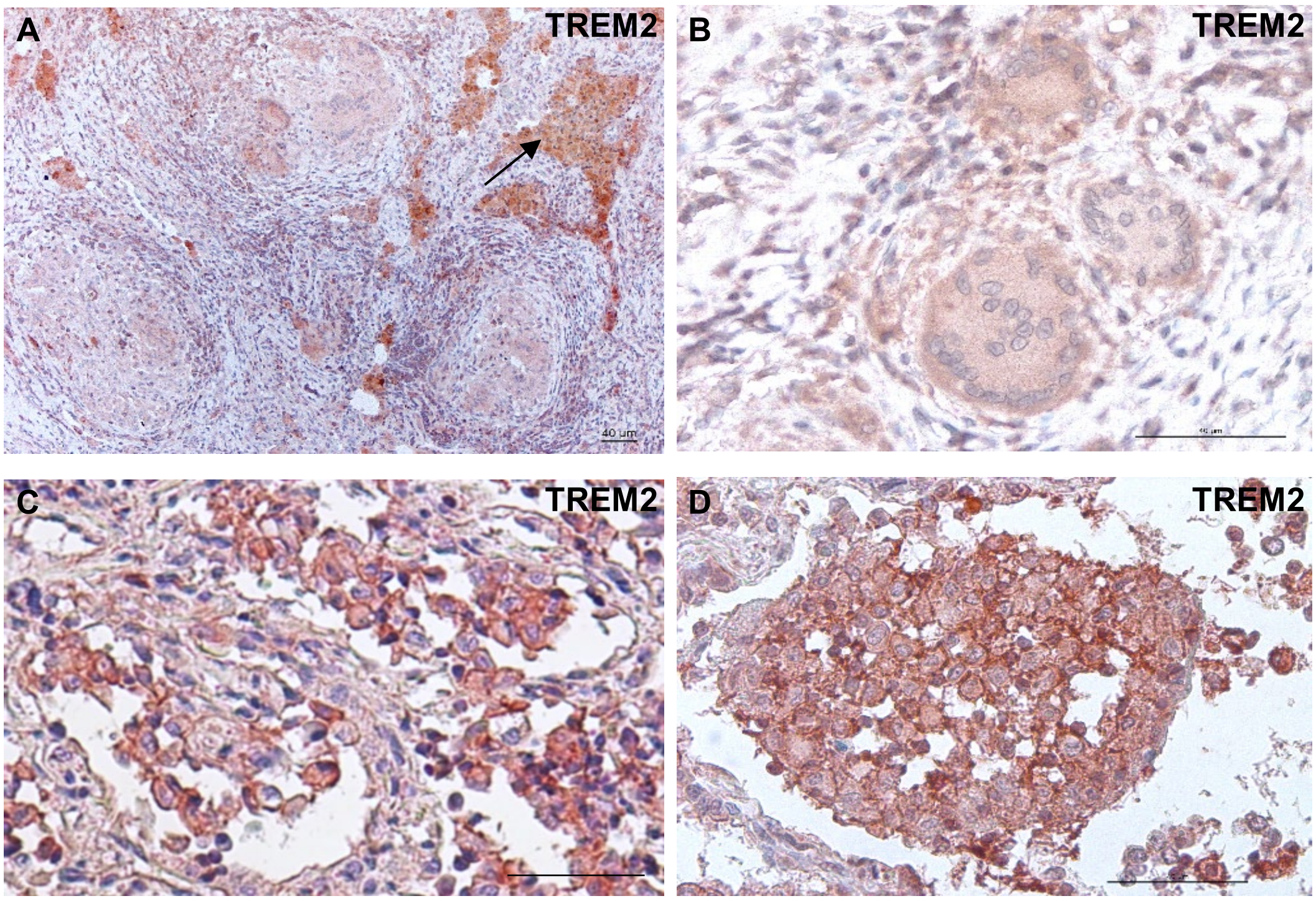

Supplementary Figure 15. TREM2 expression is enriched in alveolar foamy macrophages compared with granulomatous giant cells. Representative immunohistochemical staining for TREM2 in pulmonary tuberculosis tissue. (A) Low-power image showing TREM2 expression within PTB lesions. (B) Weak TREM2 staining is detected in multinucleated Langhans giant cells within granulomas. (C,D) Strong TREM2 expression is observed in alveolar foamy macrophages occupying surrounding alveolar spaces. Representative images are shown from four independent PTB donors (n = 4). Scale bars, 40 μm.

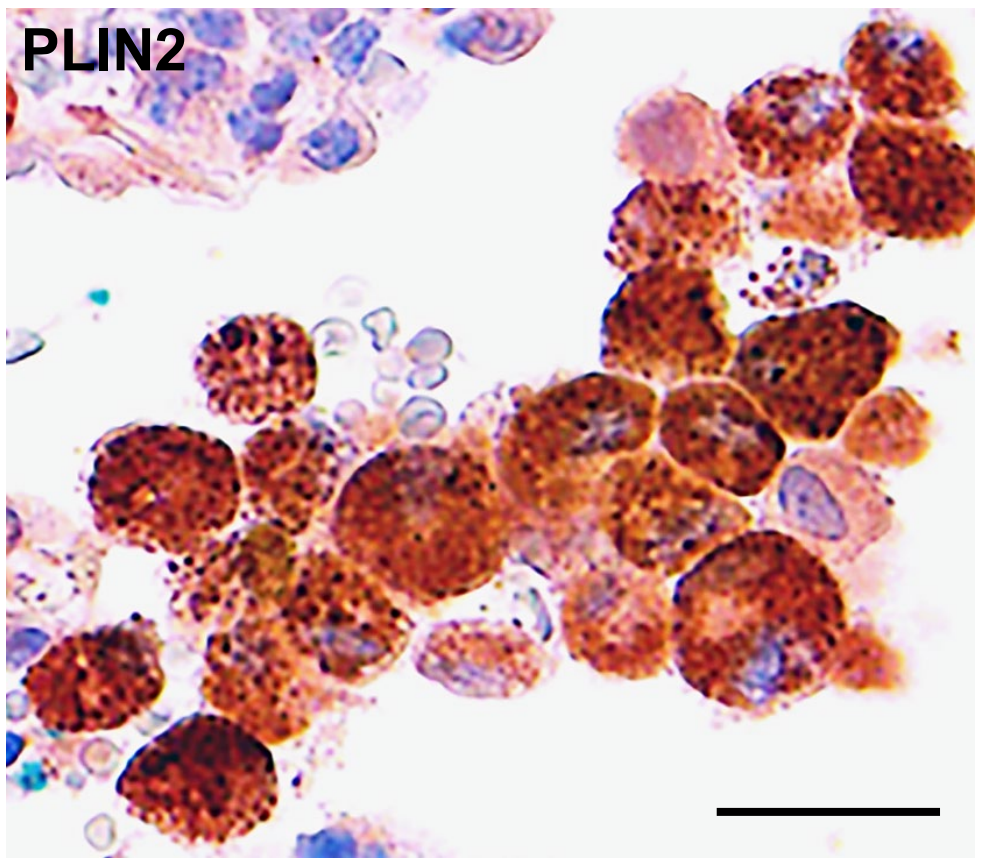

Supplementary Figure 16. PLIN2-positive lipid droplets in alveolar foamy macrophages from pulmonary tuberculosis lesions. Representative high-magnification immunofluorescence image demonstrating abundant PLIN2-positive puncta corresponding to intracellular lipid droplets within alveolar foamy macrophages. PLIN2 staining highlights the characteristic lipid-rich phenotype of these macrophages in pulmonary tuberculosis lesions. Representative images are shown from four independent PTB donors (n = 4). Scale bar, 10 μm.

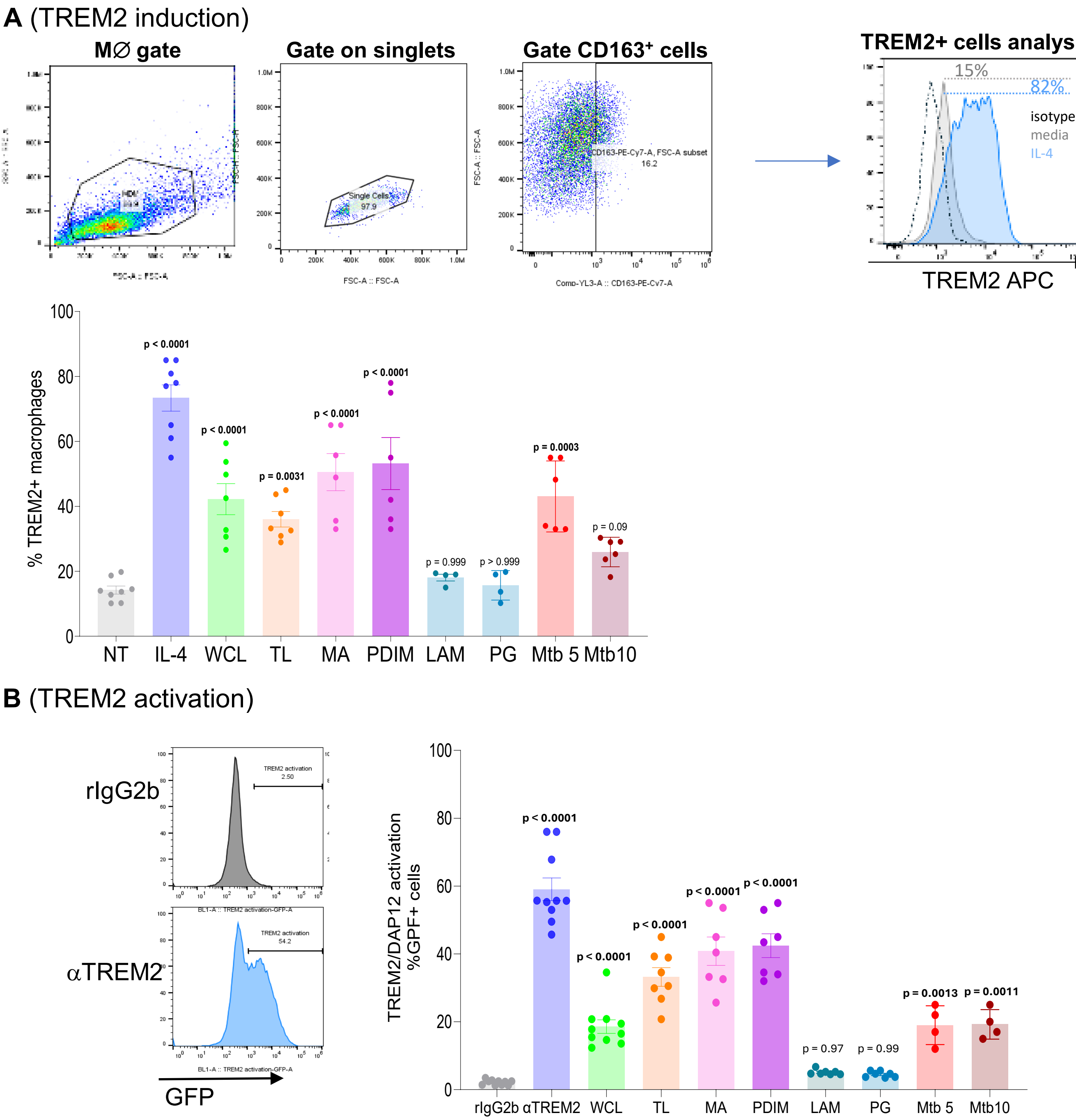

Supplementary Figure 17. TREM2 induction and activation in response to *Mycobacterium tuberculosis* (*Mtb*) ligands. (A) Human monocyte-derived macrophages (MDMs) were treated with IL-4 (100 ng/mL) or *Mtb* ligands for 48 h. TREM2 expression was assessed by flow cytometry after gating on singlets and CD163$^+$ macrophages. TREM2 gates were established using matched isotype controls, and background signal was subtracted during analysis. (B) GFP-TREM2 reporter 2B4 cells were treated for 24 h with anti-TREM2 monoclonal antibody (αTREM2), isotype control (rIgG2b), or *Mtb* ligands. GFP-positive cells, indicating TREM2/DAP12 activation, were quantified by flow cytometry. *Mtb* ligands included whole-cell lysate (WCL), total lipids (TL), mycolic acids (MA), phthiocerol dimycocerosates (PDIM), lipoarabinomannan (LAM), peptidoglycan (PG), and live *Mtb* at MOI 5 (*Mtb*5) or MOI 10 (*Mtb*10). Representative plots are shown. The number of biological replicates varied by condition (n = 4–8 independent donors). Statistical significance was determined using one-way ANOVA followed by Tukey's multiple-comparison test. Significant p values are in bold.

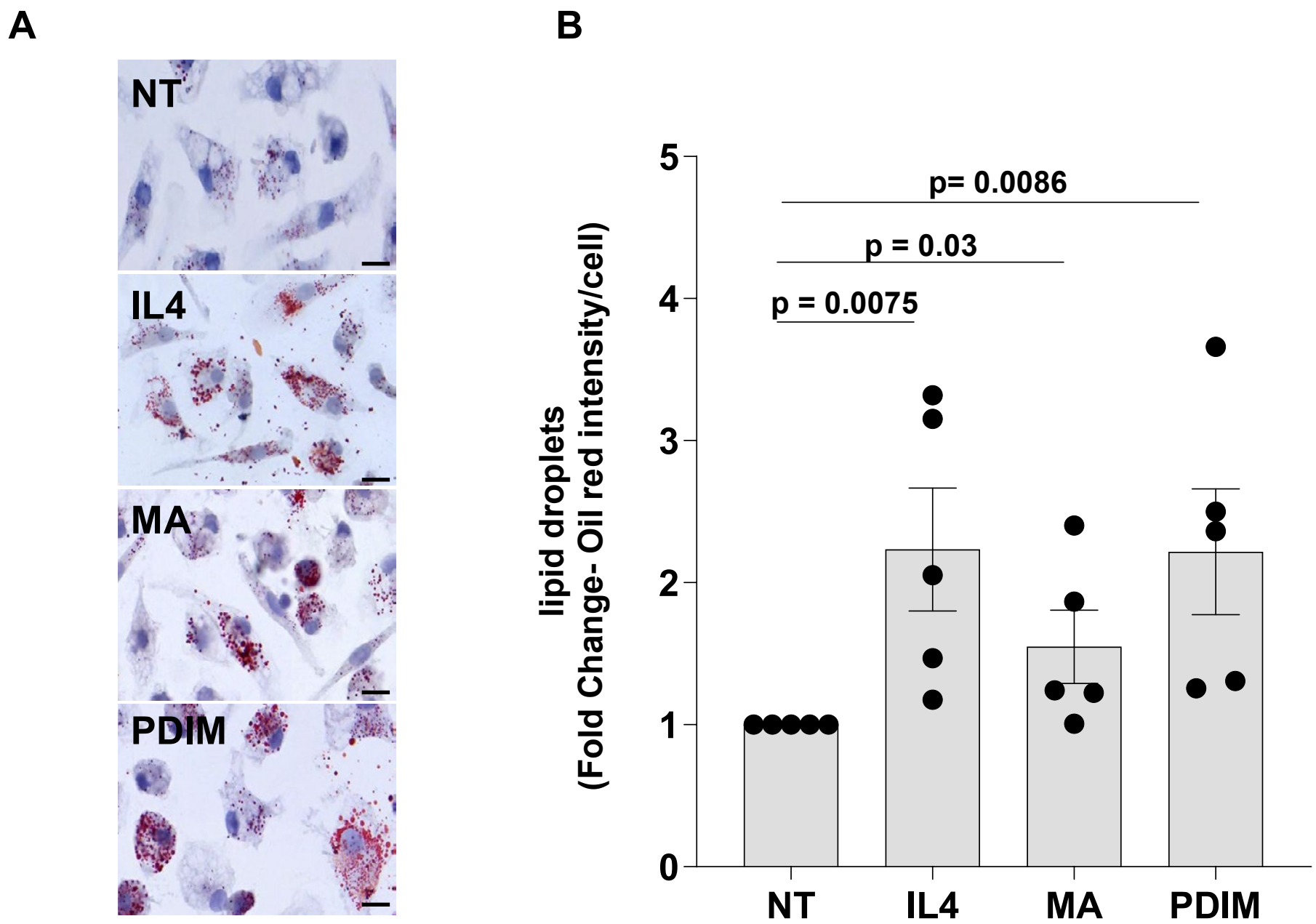

Supplementary Figure 18. Mycobacterial lipids are associated with lipid droplet accumulation in human monocyte-derived macrophages. (A) Representative Oil Red O staining of untreated macrophages (NT), IL-4-induced TREM2-positive macrophages, and macrophages stimulated with mycolic acids (MA) or phthiocerol dimycocerosates (PDIM). Both MA and PDIM are associated with increased intracellular lipid droplet accumulation resembling that observed following IL-4 polarization. Scale bar: 10 μm. (B) Quantification of lipid droplet abundance expressed as fold-change in Oil Red O-positive droplets per cell relative to untreated macrophages. Each symbol represents one independent donor (n = 5). Multiple microscopic fields were analyzed for each donor and averaged before donor-level statistical analysis. Statistical significance was determined using one-way ANOVA with Fisher's least significant difference (LSD) multiple-comparison test performed on raw lipid droplet counts prior to normalization. Data are presented as mean ± SEM.

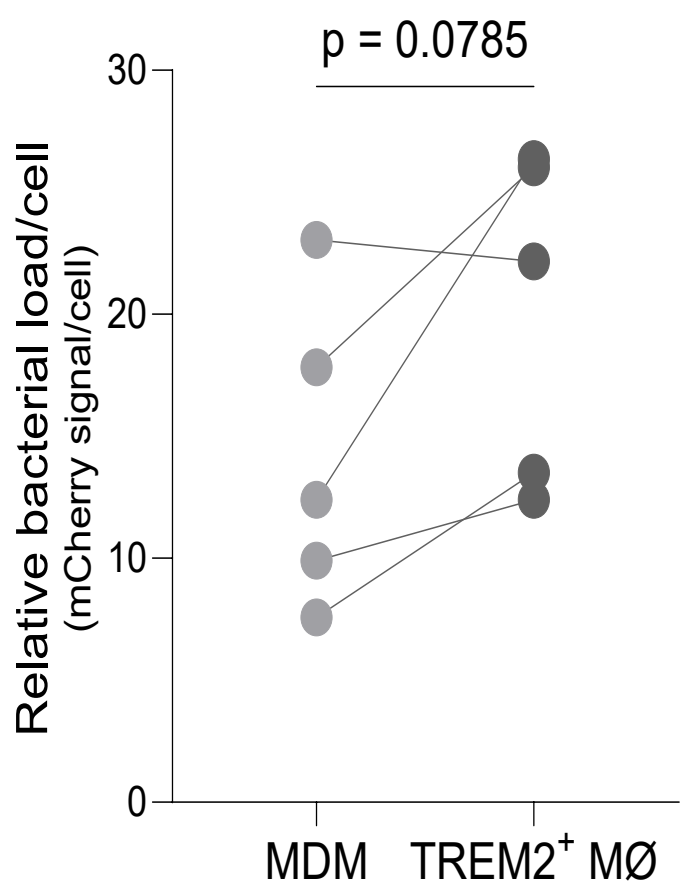

Supplementary Figure 19. Quantification of intracellular *Mycobacterium tuberculosis* (*Mtb*) burden using a dual-reporter *Mtb* strain. Human monocyte-derived macrophages (MDMs) were infected with a dual-reporter *Mtb* strain constitutively expressing mCherry and inducibly expressing GFP. Representative confocal images are shown with DAPI nuclear counterstaining. Constitutive mCherry fluorescence was quantified using ImageJ as a measure of intracellular bacterial burden. Each symbol represents one independent donor (n = 5). Multiple microscopic fields were analyzed for each donor and averaged before donor-level statistical analysis. Statistical analysis was performed using one-way ANOVA with Tukey's multiple-comparison test. Data are presented as individual values with mean ± SEM. No statistically significant differences were observed between groups.

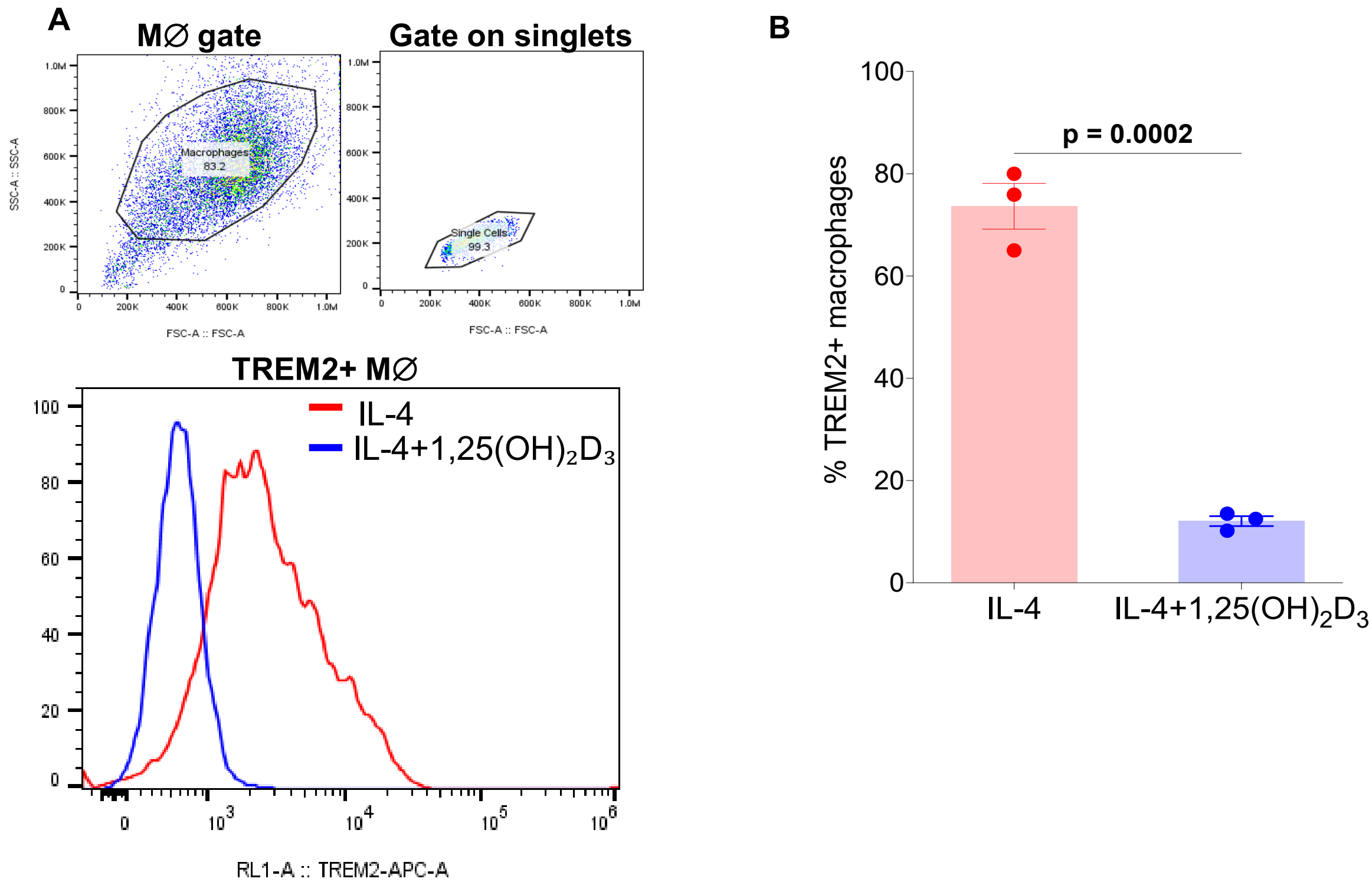

Supplementary Figure 20. (A) Representative flow cytometry plots showing TREM2 expression in IL-4-induced TREM2$^+$ macrophages treated with 1,25-dihydroxyvitamin D_3 (1,25(OH)$_2$D$_3$) for 24 h. TREM2$^+$ macrophages were gated based on FSC-A/SSC-A to identify macrophage populations. Doublets were excluded by forward scatter gating, and only viable cells were included in the analysis. Representative plots from three independent donors are shown. (B) Quantification of the percentage of TREM2-expressing macrophages following treatment with 1,25(OH)$_2$D$_3$ (n = 3 independent donors). Statistical significance was determined using one-way ANOVA with Tukey's multiple-comparison test. Data are presented as mean ± SEM.

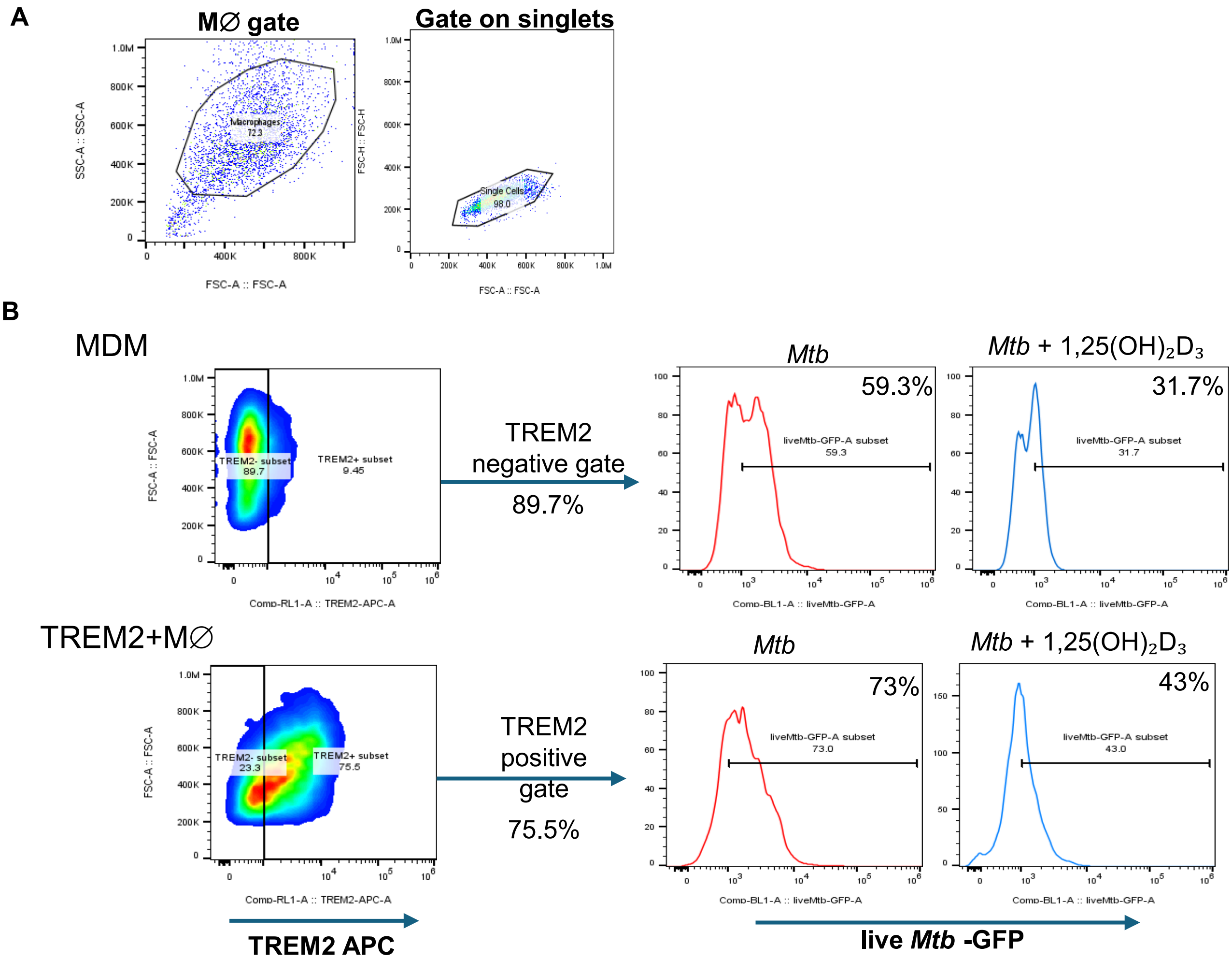

Supplementary Figure 21. Flow cytometry gating strategy for *Mtb* viability measurements shown in Fig. 7. (A) Monocyte-derived macrophages (MDMs) and IL-4–induced $TREM2^+$ macrophages were gated based on FSC-A/SSC-A to identify macrophage populations. Doublets were excluded by forward scatter gating, and only viable cells were included in the analysis. (B) TREM2 expression was used to define $TREM2^-$ and $TREM2^+$ subsets. Within each population, *Mtb* infection was assessed using a dual-reporter system, and bacterial viability was quantified based on GFP signal, corresponding to the readout used in Fig. 7. Representative histograms show the percentage of GFP^+ (live) *Mtb* in control (*Mtb*) and $1,25(OH)_2D_3$-treated conditions. Percent values indicate the fraction of viable bacteria within each gated population.

A

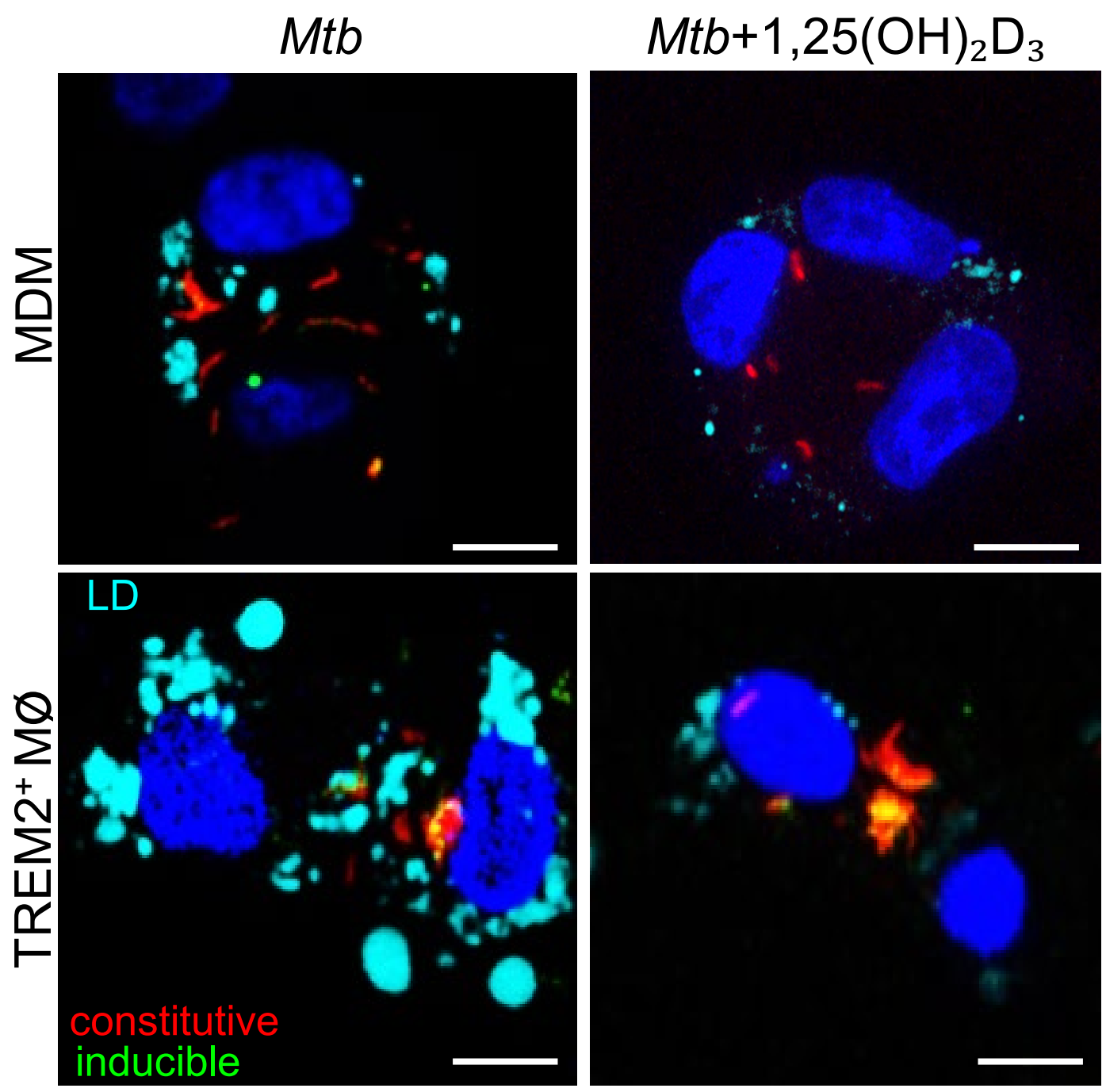

Supplementary Figure 22. Lipid droplets and *Mtb* viability are quantitatively linked in MDMs and TREM2$^+$ MØ. (A) Representative confocal images of MDMs and TREM2$^+$ macrophages infected with *Mtb* ± 1,25(OH)$_2$D$_3$, showing lipid droplets (SMCy5.5-cyan) and *Mtb* reporter signal (mCherry-constitutive/GFP-inducible). TREM2$^+$ MØ display elevated LD accumulation that is reduced by 1,25(OH)$_2$D$_3$. Representative confocal images are shown from four independent donors (n = 4). Scale bars, 10 μm.

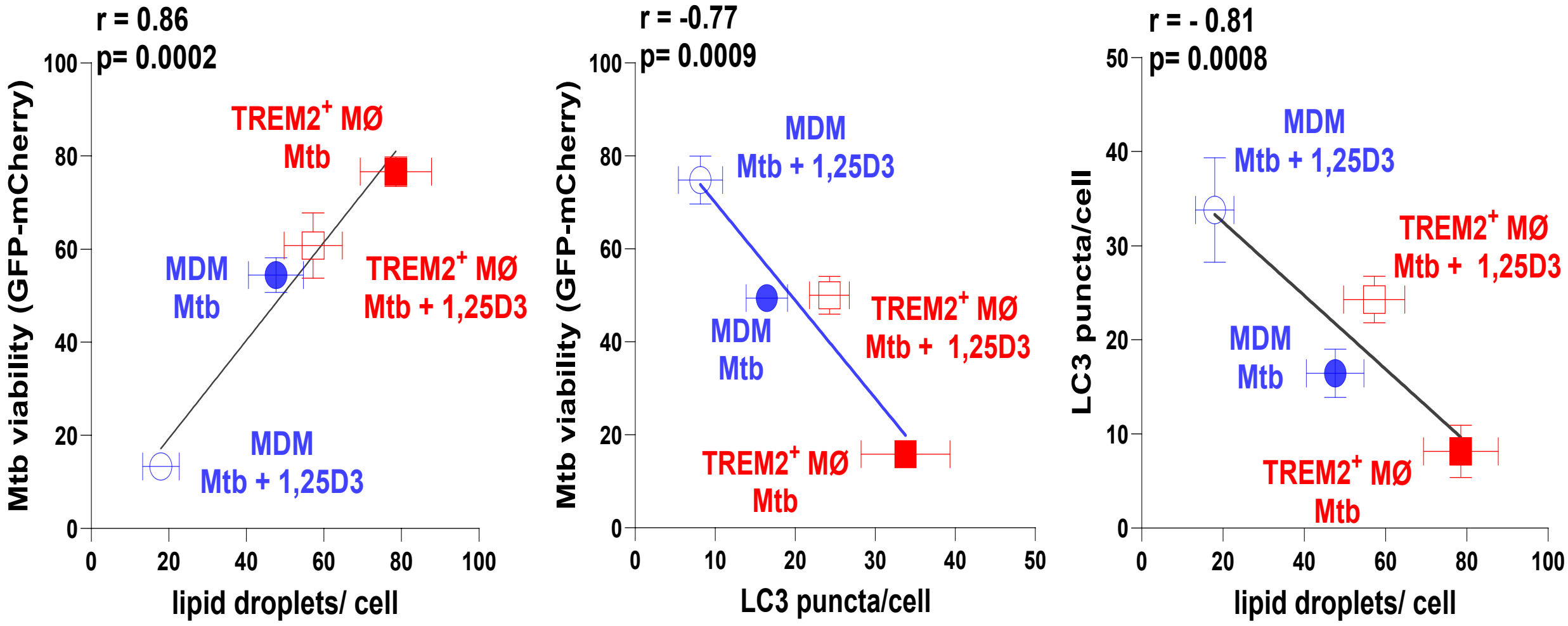

Supplementary Figure 23. Pearson correlation analysis of lipid accumulation, autophagy, and bacterial viability. Donor-matched analyses performed side by side using the same experimental samples comparing unpolarized monocyte-derived macrophages (MDM, blue) and TREM2$^+$ macrophages (red) following *Mycobacterium tuberculosis* infection, with or without 1,25-dihydroxyvitamin D_3 (1,25D3). Each symbol represents one donor. Pearson correlation analyses show positive associations between lipid droplet abundance and bacterial viability and inverse associations between LC3 puncta formation and bacterial metabolic activity. Correlation coefficients (r) and two-tailed p values were calculated as described in Methods.

Code	Age	Gender	Symptoms	Radiology	Specimen	Microbiological exam	Genexpert on FFPE	Genexpert Load of detection	Block #
**PTB-21-1**	25-30 years	Male	Smoker (10 pack-years), cessation 1 year ago,chronic cough and purulent sputum	CT scan: consistent with cavitating pulmonary TB	Lung	Not performed	Positive	Trace	B17-2914
**PTB-22-1**	30-35 years	Male	Smoker (8 pack-years), cessation 3 years ago, abundant hemoptysis	CT scan: Centrilobular nodules and micronodules	Lung	Not performed	Positive	Very Low	B19-270
**PTB-22-2**	30-35 years	Male	Non smoker, haemoptoic sputum, dyspnea, chronic cough	CT scan: Proximal nodule of the left apicoposterior segment of 18x26 mm	Lung	Not performed	Positive	Low	B19-4048
**PTB-22-3**	45-50 years	Male	Smoker (40 pack-years), chest pain	CT Scan: Mass of the right superior lobe mesuring 45 mm	Lung	Not performed	Positive	Low	B19-6077

Supplementary Table 1. Clinical and pathological characteristics of patients with pulmonary tuberculosis included in this study. Clinical information, histopathological diagnosis, and demographic characteristics of the four pulmonary tuberculosis (PTB) patients analyzed.

Patient	Annotation	Count	verage aligned	Total aligned	Nuclei per ROI	Gene detection %
PTB 21.1	Alveoli	5	93.3	33600000	777	43.8
PTB 21.1	Core	3	93.1	8470000	492	38
PTB 21.1	Mantle	3	92.2	5310000	464	33.4
PTB 22.1	Alveoli	5	94.4	24400000	109	12
PTB 22.1	Core	12	93.6	48700000	106	9.3
PTB 22.1	Mantle	5	94.2	15500000	112	9.9
PTB 22.2	Alveoli	6	92.7	45400000	93	12.9
PTB 22.2	Core	5	93.7	73800000	97	19.1
PTB 22.2	Mantle	1	93.3	6820000	81	14.5
PTB 22.3	Alveoli	4	94.8	65000000	108	25.7
PTB 22.3	Core	5	90.2	31000000	114	19.6
PTB 22.3	Mantle	5	94.2	21000000	107	16.8
Total	All	59	93.3	379000000	200	19.1

Supplementary Table 2. Quality control metrics for GeoMx spatial transcriptomic profiling. Summary of regions of interest (ROIs), sequencing metrics, alignment statistics, nuclei counts, and gene detection rates for each PTB sample included in the GeoMx analysis.

Disease	Cluster																		
	**0**	**1**	**2**	**3**	**4**	**5**	**6**	**7**	**8**	**9**	**10**	**11**	**12**	**13**	**14**	**15**	**16**	**17**	**18**
**pulmonary sarcoidosis**	0	0.006	0	0.94382	0.011	0.007	0.004	0	0.021	0.001	0.004	0	0.002	0	0	0	0	0	0
**pulmonary fibrosis**	0.305	0.033	0.462	0.138769	0.046	6E-04	9E-04	0.011	5E-04	2E-04	1E-04	######	5E-04	2.17E-05	0.000357	2E-04	1E-04	0	0
**lung large cell carcinoma**	0	0.045	0	0	0.93	0.001	0.005	0.015	5E-04	0	0	0.0023	9E-04	0	0.000939	0	0	0	0
**pleomorphic carcinoma**	0	0.154	0	0	0.311	0	0.006	0.09	0	0.004	0	0.0019	0	0	0	0.434	0	0	0
**chronic rhinitis**	0	0	0	0	0.689	0.006	0.037	0.093	0	0	0.174	0	0	0	0	0	0	0	0
**chronic obstructive pulmonary disease**	0.905	0	0.095	0	0	0	0	0	0	2E-04	0	0	0	0	0	0	0	0	0
**lung adenocarcinoma**	0	0.276	0	0.000468	0.65	0.001	0.013	0.042	0	2E-04	9E-04	0.0017	0.005	0.000624	0.003121	0.001	0.005	0	0
**squamous cell lung carcinoma**	0	0.497	0	0	0.438	0.002	0.009	0.042	4E-04	0	8E-04	0.0044	0.003	0	0.002482	9E-04	1E-04	0	0
**pneumonia**	0	2E-04	0	6.97E-05	6E-04	0.008	0.002	0.795	0.089	6E-04	0.088	0.0111	0.003	0.003274	0	0	0	0	0
**lymphangioleiomyomatosis**	0	0.93	0	0	0.061	2E-04	0	6E-04	0	2E-04	0	0.0004	0	0	0.001487	0.005	0	0	0
**interstitial lung disease**	0	0.268	0	0.059338	0.296	0.004	0.339	0.026	3E-04	1E-04	0.002	0.0006	0.004	9.07E-05	0.000317	0.001	4E-04	0	0
**hypersensitivity pneumonitis**	0	0.164	0	0.756239	0.076	3E-04	0	0	0.001	0	0	0	0	0	0.000504	0.001	3E-04	0	0
**non-specific interstitial pneumonia**	0	0.003	0	0.979385	0.005	0	1E-03	7E-04	0.008	3E-04	0.002	0.0007	0	0	0	0	3E-04	0	0
**COVID-19**	0	6E-04	0	8.20E-05	0.002	0.006	0.315	0.042	0.059	0.115	0.28	0.0015	0.003	0.174538	4.92E-05	0	2E-04	1.64E-05	0.0006
**normal**	0.083	0.184	0.076	0.108847	0.069	0.139	0.003	0.038	0.074	0.048	0.002	0.0628	0.054	9.32E-05	0.026174	0.017	0.014	0.001872	0

Supplementary Table 3. Analysis of TREM2-associated macrophage signatures in the Human Lung Cell Atlas. Macrophage cluster annotation and disease distribution.

leiden_v1	n_cells	TREM2_expr
**0**	74392	0.51342773
**1**	71104	0.83539414
**2**	66249	0.78081363
**3**	51993	1.1468353
**4**	40483	0.96513504
**5**	39383	0.9034417
**6**	27845	0.8898056
**7**	26814	0.5388617
**8**	25731	1.1272204
**9**	20561	0.1461801
**10**	19038	1.01838
**11**	17826	1.170983
**12**	15341	0.86421704
**13**	10728	0.57027215
**14**	7392	1.0407791
**15**	4961	0.9711276
**16**	4054	0.15509231
**17**	523	1.2209815
**18**	34	0.16421862

Supplementary Table 4. Analysis of TREM2-associated macrophage signatures in the Human Lung Cell Atlas. Average TREM2 expression across macrophage clusters.

leiden	TREM2 genes in 20+ papers	TREM2 genes in 12+ papers	PTB TREM2 macrophages
0	0.645590006	0.780018871	0.393870171
1	0.945686565	1.210806657	0.583086704
2	0.636282511	0.824567981	0.455097249
3	0.943985268	1.227721774	0.616053932
4	0.901287685	1.174464492	0.621431739
5	0.850240186	1.198964677	0.60625441
6	1.011314096	1.259278879	0.76388969
7	0.817260284	0.876170397	0.438007191
8	1.06800048	1.250279371	0.637355857
9	0.181227406	0.371309236	0.282084213
10	0.764837238	1.130433625	0.583933705
11	1.184089468	1.310121974	0.638854896
12	0.806636137	1.171776168	0.57891755
13	0.854814076	1.166759561	0.601283103
14	0.954856978	1.25421837	0.569451728
15	0.914786885	1.239253066	0.61657231
16	0.818574584	1.161532029	0.561186547
17	0.891112285	1.208326524	0.799452667
18	1.22473231	0.994286594	0.455028181

Supplementary Table 5. Analysis of TREM2-associated macrophage signatures in the Human Lung Cell Atlas. TREM2-associated signature scores across macrophage clusters.

Disease	Citation	Finding	Reported frequency in diseased lung	Metric / denominator	Direct quotation from source	Compartment	Method for localization	Healthy lung	Where reported	Frequency note
COPD	Byers DE, TREM-2 expression tracks with macrophage activity in COPD, Chest 2018	TREM2⁺ tracks with M2-like macrophage activation	No percentage reported; TREM2/TREM1 mRNA ratio and TREM-2 protein were increased in COPD lung tissue.	mRNA/protein expression; CD14+, CD68+, and CCR2+ macrophage localization	"TREM-2 expression is a robust marker of lung tissue macrophage populations"	diffuse lung macrophages (not compartment-resolved)	immunofluorescence + flow cytometry	non-COPD lung tissue (low TREM2 expression; not strictly healthy)	Byers et al. 2018; Results, Fig. 1-3	COPD evidence is expression/localization-based. The paper does not give a clean frequency of TREM2+ macrophages in diseased lung.
pulmonary cancer and fibrosis	Mulder K, Cross-tissue single-cell landscape of human monocytes and macrophages, Immunity 2021	TREM2⁺ macrophages have a conserved lipid/damage-associated program	Not numerically reported in the text; qualitative enrichment of TREM2_Mac (#3) in lung tumors.	MoMac-VERSE TREM2_Mac (#3) cluster; diseased lung cancer/tumor datasets	"TREM2_Mac (#3) also accumulated but only in the tumors"	not determined	scRNA-seq	rare, very low	Mulder et al. 2021; text discussing lung cancer MoMac-VERSE / Fig. 4	No exact percentage was printed in the paper text available here; report as qualitative enrichment rather than a numerical frequency.
pulmonary cancer	Zhang H, Immunosuppressive TREM2(+) macrophages are associated with undesirable prognosis, Cancer Immunol Immunother 2022	TREM2⁺ tumor-associated macrophages suppress immunity	Estimated from Fig. 1F: intratumoral TREM2+ TAMs are roughly ~55-60% of CD68+ TAMs, versus ~20-30% in peritumor tissue; exact mean not printed.	% of CD68+ TAMs in NSCLC tumor vs peritumor tissue by flow cytometry	"substantially accumulate in tumor tissues rather than peritumor tissues"	tumor tissue (no microanatomic resolution)	immunofluorescence + flow cytometry	low frequency in adjacent tissue	Zhang et al. 2022; Results and Fig. 1F	The numerical value is estimated from the plotted bar graph. The text states accumulation but does not provide a printed mean ± SD.
pulmonary fibrosis	Fabre T, Identification of a broadly fibrogenic macrophage subset induced by type 3 inflammation, Sci Immunol 2023	CD9⁺TREM2⁺ macrophages represent fibrogenic macrophage state	Estimated from figures: Fab5 SAMs roughly ~15% cell abundance in fibrotic/IPF lung by scRNA-seq Fig. 2C; roughly ~60% SPP1+GPNMB+FABP5+ by cycIF Fig. 2G.	Fab5 SAM signature; CD9+TREM2+SPP1+ / SPP1+GPNMB+FABP5+ macrophages; graphical estimates	"CD9+TREM2+SPP1+ macrophages increased in both liver and lung fibrosis"	fibrotic scar edge (interstitium)	immunofluorescence + cycIF	low frequency	Fabre et al. 2023; Results and Fig. 2C/G	The paper reports frequencies graphically; exact numerical means were not printed in the article text. Values should be treated as approximate graph-read estimates.
SARS-CoV-2	Upadhyay AA, TREM2+ and interstitial-like macrophages orchestrate airway inflammation in SARS-CoV-2 infection in rhesus macaques, Nat Commun 2023	TREM2⁺ macrophages are recruited inflammatory airway macrophages	At 4 dpi BAL: pooled CD163+MRC1+TREM2+ macrophages increased to 36.8% in Cohort 1 and 19.8% in Cohort 2; individual-animal means were 28.6% and 20.0%.	% of all BAL macrophages/monocytes; rhesus macaque SARS-CoV-2 infection	"CD163+MRC1+TREM2+ macrophages from 6.5 to 36.8% (Cohort 1) and 10.3 to 19.8% (Cohort 2)"	alveolar/airway compartment	BAL + scRNA-seq (no histologic localization)	low frequency at baseline	Upadhyay et al. 2023; Results and Fig. 3e-h	This is the clearest numeric diseased-lung/airway frequency in the set; denominator is BAL macrophages/monocytes.
pulmonary fibrosis	Cui H, TREM2+promotes lung fibrosis via controlling alveolar macrophage survival, Nat Commun 2025	TREM2⁺ expressed on monocyte-derived alveolar macrophages driving fibrosis	No TREM2+ macrophage percentage reported; increased TREM2 expression/MFI in fibrotic lung macrophages and IPF AMs.	Expression/MFI, not frequency; Mo-AMs / IPF alveolar macrophages	"markedly increased TREM2 expression in IPF AMs compared to normal lungs"	alveoli	immunofluorescence	sparse	Cui et al. 2025; Results, Fig. 1D/E and Fig. 2C	Use this study for localization/expression, not frequency. It supports TREM2-high Mo-AMs in fibrotic lung.

Supplementary Table 6. Published studies describing TREM2-associated macrophages in pulmonary diseases. Summary of studies reporting TREM2-associated macrophage populations across human lung diseases.

	Alveoli	Core	Mantle	AvM	MvA	CvM	MvC	AvC	CvA
# Antimicrobial genes	3	8	5	5	10	9	6	3	15
Jaccard overlap	0.004942	0.017505	0.005889	0.004026	0.007326	0.008687	0.005063	0.003099	0.014437
**Adj p value**	0.251715	6.78E-05	0.120881	0.25477	0.006134	0.004001	0.133424	0.462776	3.72E-07
**Antimicrobial Genes**	CXCL2	IL1B	CCL21	CXCL2	CCL21	IL1B	CCL21	CXCL2	IL1B
	CCL18	CCL1	PRF1	S100A9	PRF1	CCL1	PRF1	CCL18	CCL1
	NPC2	P2RX7	GZMB	CCL18	CXCL9	P2RX7	GNLY	NPC2	CCL17
		CCL8	CCL19	NPC2	GZMB	CCL8	GZMB		CXCL9
		TXN	CXCL13	CCL13	IL32	TXN	CCL19		IL32
		LYZ			GBP1	LYZ	CXCL13		GBP1
		CAMP			CCL19	CAMP			P2RX7
		IDO1			CXCL13	IDO1			CCL8
					CD40LG	S100A8			CCL19
					IDO1				VDR
									TXN
									LYZ
									CAMP
									CD40LG
									IDO1

Supplementary Table 7. Overlap of antimicrobial gene signatures with pulmonary tuberculosis spatial and single-cell transcriptomic datasets. Comparison of curated antimicrobial gene signatures with GeoMx spatial transcriptomic and scRNA-seq datasets**.**
